# Transient blood-brain barrier opening by focused ultrasound enhances ferumoxytol accumulation in glioblastoma

**DOI:** 10.1016/j.mtbio.2026.103280

**Published:** 2026-05-26

**Authors:** Giovanni M. Saladino, Payton J. Martinez, Jie Wang, Raheleh Roudi, Giacomo Annio, Raag D. Airan, Heike E. Daldrup-Link

**Affiliations:** Department of Radiology, School of Medicine, Stanford University, Stanford, CA, 94305, USA

**Keywords:** Brain tumor barrier modulation, Ferumoxytol delivery, Magnetic resonance imaging, Glioblastoma, Microbubble-mediated focused ultrasound

## Abstract

Nanoparticle delivery to glioblastoma is limited by the blood-brain barrier (BBB), which restricts the transport of diagnostic and therapeutic agents into tumor tissue. Microbubble-mediated focused ultrasound (FUS) can transiently increase BBB permeability and enhance localized delivery of molecular probes and drugs. We tested whether MRI-guided microbubble-assisted FUS at 650 kHz enables spatially controlled and quantitatively measurable delivery of the FDA-approved ferumoxytol (FMX), an iron oxide nanoparticle formulation. Acoustic simulations were employed to quantify transcranial pressure fields and assess pressure-dependent BBB opening at a clinically relevant frequency and were experimentally validated in healthy mice. In mice bearing orthotopic U87 glioblastoma, MRI-guided microbubble-assisted FUS was focally applied to the tumor region, with intravenously administered Definity microbubbles mediating transient BBB opening, followed by intravenous injection of fluorescein-labeled FMX. Nanoparticle accumulation was quantified using *in vivo* MRI and *postmortem* fluorescence imaging. Post-contrast tumor T_2_ relaxation times were 33.14 ms in the FMX group and 22.47 ms in the FMX + FUS group. Tumor transverse relaxation rate change showed a 2.6-fold increase under FUS conditions relative to the non-FUS group, demonstrating enhanced nanoparticle transport across a transiently permeabilized BBB. Pressure thresholds were identified for FMX delivery to normal brain versus tumor tissue, with a lower estimated threshold in tumor tissue than in healthy brain (0.224 MPa vs. 0.265 MPa), highlighting a pressure window for selective nanoparticle transport. Histopathological evaluation of H&E-stained normal brain and tumor sections showed no significant FUS-associated increase in detectable acute structural tissue damage. Our findings establish a quantitative and clinically translatable framework for spatially controlled FUS-enhanced delivery of clinically relevant nanoparticle formulations to brain tumors, advancing targeted delivery strategies for neuro-oncologic applications.

## Highlights

• Quantitative framework for MRI-guided FUS nanoparticle delivery.
• Clinically relevant transcranial FUS enhances ferumoxytol delivery.
• MRI tracks FUS-enhanced tumor accumulation *in vivo.*
• Pressure thresholds quantified for tumor-selective delivery.

## Graphical abstract

**Figure:**
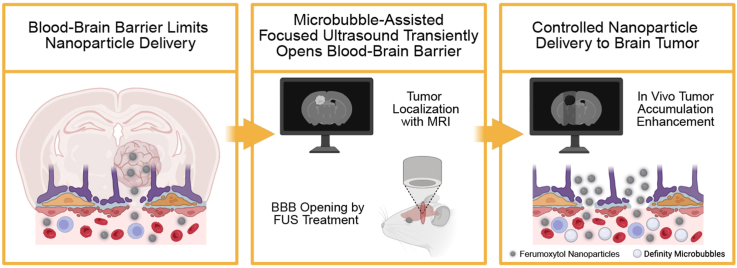

## 1 Introduction

Nanoparticle-based strategies for diagnosis and therapy have transformed many areas of oncology, yet their application in brain tumors remains substantially limited by the restrictive nature of the blood-brain barrier (BBB). The BBB hinders the entry of most systemically administered molecular and nanoparticle agents into the central nervous system [1,2]. Even nanoparticles engineered for enhanced transport often achieve only marginal brain accumulation. Wilhelm et al. found that only 0.7% (median) of systemically administered dose of nanoparticles is delivered into solid tumors, thus limiting their utility in clinical settings where reliable tumor contrast or therapeutic efficacy is needed [3].

A growing body of research has focused on developing methods to enhance nanoparticle access to the brain. For example, strategies that exploit receptor-mediated transcytosis or disease microenvironment features have shown variable improvements in nanoparticle transport but remain limited by low delivery efficiency and poor clinical translation. Hynynen et al. developed an alternative approach, employing focused ultrasound (FUS) complemented by microbubbles as a noninvasive method to transiently and locally disrupt the BBB or blood-tumor barrier (BTB) [4]. These microbubbles are generally 1-10 μm in diameter and consist of a stabilizing shell (*e.g.*, lipid or protein) surrounding a gas core (*e.g.*, perfluorocarbon) [5]. Under exposure to low-frequency ultrasound (0.2-2 MHz), the circulating gas-filled microbubbles undergo pronounced oscillation compared with the surrounding fluid and viscoelastic tissue. The resulting localized mechanical stresses lead to transient separation of endothelial tight junctions, thereby disrupting the BBB/BTB [6,7]. Clinical studies have demonstrated that FUS-mediated BBB/BTB opening is well tolerated, with no evidence of significant neuronal injury, apoptosis, ischemia, or lasting vascular damage [[8], [9], [10]]. The induced BBB/BTB opening is spatially confined and transient, persisting for approximately 3 to 24 h depending on the magnitude of mechanical stress, which is governed by ultrasound parameters and microbubble dosage [11].

In preclinical glioma models, Wei et al. demonstrated that FUS-induced BBB/BTB opening increases delivery of temozolomide, and Martinez et al. reported similar enhancements for panobinostat, with both studies showing improved tumor control and survival [12,13]. Similarly, FUS has been shown to enhance the delivery of etoposide in orthotopic glioblastoma, suggesting broader applicability for chemotherapeutic agents when combined with ultrasound-mediated BBB/BTB opening [14]. Importantly, these studies were conducted at ultrasound frequencies of 1 MHz or higher, which are well suited for murine models due to the thin mouse skull (∼0.5 mm) and relatively low acoustic attenuation [15]. In contrast, the human skull is substantially thicker (∼5-8 mm) with a thicker diploic space, resulting in markedly higher attenuation (90% in human *vs*. 18% in mouse at 1 MHz) and significant phase aberration [16,17]. As a result, these frequencies pose challenges for efficient and predictable transcranial delivery in humans, limiting direct clinical translation. Accordingly, clinical BBB/BTB opening protocols more typically utilize sub-MHz ultrasound, generally in the 220-650 kHz range.

In addition, the therapeutic agents evaluated in these studies are small-molecule chemotherapeutics (<1 kDa), which readily diffuse across the transient BBB openings induced by FUS. By comparison, nanoparticle-based platforms often range from 10 to 100 nm in diameter and therefore exhibit fundamentally different transport constraints. Nance et al. investigated delivery of gold nanoparticles with varying diameters across microbubble-mediated FUS (1.14 MHz) BBB opening models and found that optimal delivery *in vivo* depended on a balance between BBB permeation and systemic clearance [18]. Other efforts applying FUS to nanoparticle delivery often rely on surrogate tracers, liposomal carriers, or polymeric formulations, and rarely integrate *in vivo* imaging [11,19]. For example, Liu et al. demonstrated microbubble-mediated focused ultrasound delivery of quercetin-loaded sulfur nanoparticles across the BBB for neuroprotective therapy in Alzheimer's disease [20], and Ogawa et al. showed that microbubble-mediated FUS enables lipid nanoparticle-assisted mRNA delivery to the brain for transient gene expression [21]. While FUS-mediated BBB opening has been shown to permit passage of macromolecules up to several tens of nanometers under select conditions, these effects are highly dependent on acoustic parameters and experimental context. Systematic demonstration of efficient and reproducible delivery of clinically relevant nanoparticle systems, particularly under acoustic conditions compatible with human transcranial application, remains limited. Moreover, FUS-mediated delivery of nanoparticles to brain tumors using clinically relevant FUS frequencies and direct *in vivo* nanoparticle tracking has not been performed yet.

Ferumoxytol (FMX) is an iron-based nanoparticle formulation, recently FDA-approved as an MRI contrast agent for brain tumor detection [22]. As a non-gadolinium alternative composed of superparamagnetic iron oxide nanoparticles, FMX is particularly suitable for patients with renal insufficiency. Furthermore, its carboxymethyldextran coating supports chemical functionalization for drug loading, establishing FMX as a highly versatile, clinically applicable nanotheranostic platform [[23], [24], [25], [26]]. Thus, FMX provides a clinically relevant nanoparticle probe that can be directly quantified by MRI, rather than relying on a separate surrogate tracer.

In our study, we investigate whether BBB/BTB opening by microbubble-mediated FUS at a clinically relevant frequency (650 kHz) can enhance tumor uptake of FMX in a mouse model of glioblastoma in a spatially controlled and quantitatively measurable manner. Conjugation of FMX with fluorescein isothiocyanate (FITC) was performed to provide an *ex vivo* fluorescence readout complementary to MRI, enabling a dual modality approach for quantitative *in vivo* tracking with MRI and *ex vivo* fluorescence imaging after tissue harvest. Predictive simulations and studies with healthy mice were performed to validate FUS at 650 kHz. We then hypothesized that microbubble-mediated FUS treatment transiently disrupts the BTB and produces an increase in FMX tumor accumulation compared with untreated tumor tissue. The purpose of this study is to establish proof of concept for a clinically translatable approach to overcome BTB limitations and enhance nanoparticle delivery in glioblastomas, while defining pressure thresholds for nanoparticle accumulation in tumor and healthy brain tissue as a quantitative basis for selective nanoparticle delivery.

## 2 Results

To evaluate FUS-mediated delivery of FMX to glioblastoma, we characterized the employed dual-mode FMX nanoparticles, validated pressure-dependent BBB opening in healthy brain, and quantified enhanced tumor accumulation of FMX using MRI and fluorescence imaging.

### 2.1 Dual-mode ferumoxytol nanoparticles

**Fig. 1:**
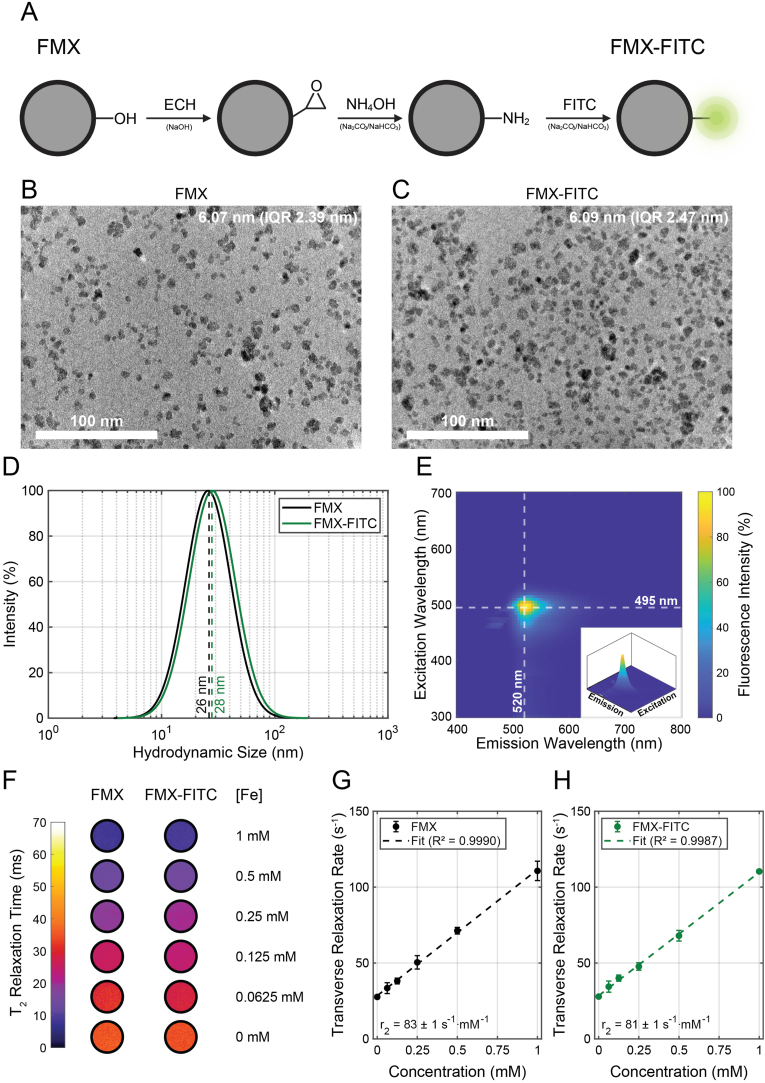
Fluorescent Ferumoxytol. (**A**) Scheme of the conjugation steps of Ferumoxytol (FMX) with Fluorescein Isothiocyanate (FITC). The FMX surface (-OH) is reacted with epichlorohydrin (ECH) under alkaline (NaOH) conditions, leading to the formation of an epoxide ring, which is subsequently modified by ammonium hydroxide (NH_4_OH) in bicarbonate buffer to achieve aminolysis. The resulting primary amine is then reacted with FITC to yield FMX-FITC. (**B**) Transmission electron micrograph of FMX showing quasi-spherical nanoparticles with an estimated dry size of 6.07 nm (IQR 2.39 nm). (**C**) Transmission electron micrograph of FMX-FITC showing quasi-spherical nanoparticles with an estimated dry size of 6.09 nm (IQR 2.47 nm). (**D**) Dynamic light scattering measurements of FMX (black) and FMX-FITC (green), highlighting their median hydrodynamic sizes of 26 nm and 28 nm, respectively. (**E**) 2D fluorescence spectrum of FMX-FITC highlighting the excitation and emission maxima (dashed white lines) at 495 nm and 520 nm, respectively. The inset shows a 3D view of the fluorescence peak. (**F**) T_2_ relaxation time measurements of agar phantoms containing different concentrations of FMX and FMX-FITC, showing a concentration-dependent response. (**G**) Transverse relaxation rate of FMX as a function of iron concentration, showing a linear correlation (R^2^ = 0.9990, P < 0.001), yielding a transverse relaxivity of 83 ± 1 s^−1^ mM^−1^. (**H**) Transverse relaxation rate of FMX-FITC as a function of iron concentration, showing a linear correlation (R^2^ = 0.9987, P < 0.001), yielding a transverse relaxivity of 81 ± 1 s^−1^ mM^−1^. (For interpretation of the references to color in this figure legend, the reader is referred to the Web version of this article.)

FITC labeling was employed to enable *ex vivo* optical validation of FMX accumulation and to demonstrate the chemical functionalizability of FMX. FMX underwent epoxidation, aminolysis, and subsequent reaction with FITC (Fig. 1A). Transmission electron microscopy showed quasi-spherical nanoparticles for both FMX and FMX-FITC (Fig. 1B–C).

Core diameter distributions derived from TEM images were fitted with lognormal functions (Fig. S1). FMX displayed a dry core median diameter of 6.07 nm (IQR 2.39 nm), while FMX-FITC showed a median diameter of 6.09 nm (IQR 2.47 nm), with goodness-of-fit values of R^2^ = 0.95 and R^2^ = 0.96, respectively. No significant difference was observed between the two distributions (P = 0.4774). Dynamic light scattering measurements showed monomodal size distributions and indicated hydrodynamic diameters of 26 nm for FMX and 28 nm for FMX-FITC (Fig. 1D). The polydispersity indices were 0.23 for FMX and 0.24 for FMX-FITC.

The 2D fluorescence spectrum of FMX-FITC showed an excitation maximum at 495 nm and an emission maximum at 520 nm (Fig. 1E, Fig. S2–S3, cf. Supplementary Text) [23].

T_2_ relaxation times of agar phantoms containing increasing concentrations of FMX and FMX-FITC are shown in Fig. 1F. Transverse relaxation rates (R_2_) were plotted as a function of iron concentration (Fig. 1G–H). Linear regression yielded R^2^ = 0.9990 (P < 0.001) for FMX and R^2^ = 0.9987 (P < 0.001) for FMX-FITC. The transverse relaxivities were 83 ± 1 s^−1^ mM^−1^ for FMX and 81 ± 1 s^−1^ mM^−1^ for FMX-FITC. No significant difference in slopes was observed (P = 0.3662).

### 2.2 Focused ultrasound simulations

**Fig. 2:**
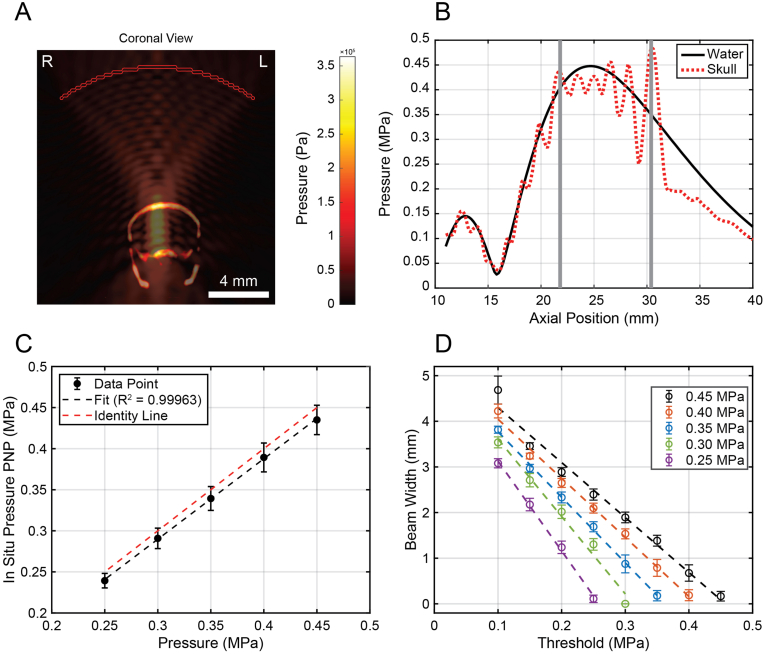
Simulation Studies of Focused-Ultrasound Treatment. (A) Coronal view of the simulated focused ultrasound pressure field overlaid on mouse brain CT data. (**B**) Centerline pressure profiles comparing simulated pressure fields in water (black) and through the skull (red). Grey vertical dashed lines indicate the superior and inferior boundaries of the skull. (**C**) *In situ* peak negative pressure (PNP) as a function of applied acoustic pressure for five simulated pressure levels. The dashed black line shows the linear regression (R^2^ = 0.99963), and the dashed red line indicates the identity line (y = x). The regression was significantly different from the identity line (joint test H_0_: slope = 1 and intercept = 0, P = 0.002). (**D**) Estimated beam width as a function of threshold pressure for five simulated acoustic pressure levels. Dashed lines of the corresponding colors indicate linear regression fits for each simulated pressure condition, with R^2^ > 0.98 and P < 0.005 for all fits. (For interpretation of the references to color in this figure legend, the reader is referred to the Web version of this article.)

The k-Wave simulation framework was first validated against experimental hydrophone calibrations of the 650 kHz single-element FUS transducer in water (Fig. S4–S5, cf. Supplementary Text) [27]. Simulations revealed pronounced standing wave patterns within the cranial cavity arising from reflections at acoustic impedance mismatches across skull and tissue interfaces (Fig. 2A, Fig. S6) [28]. Quantitatively, the peak-to-mean pressure ratio within the brain was 1.19 ± 0.12 (N = 6), indicating reproducible but spatially heterogeneous pressure amplification driven by interference effects. Inter-animal variability in pressure modulation, quantified by the coefficient of variation, was 0.13 ± 0.05, further supporting consistent standing-wave behavior across skull geometries. The standing wave ratio, defined as the ratio of peak intracranial pressure to the expected transmitted pressure in the absence of interference, was 1.34 ± 0.20 (N = 4; two datasets excluded due to undefined values), indicating partial pressure recovery within the cranial cavity despite transcranial attenuation (Fig. 2B).

For applied peak negative pressures (PNPs) in water ranging from 0.25 to 0.45 MPa, the estimated *in situ* PNP exhibited a strong linear relationship (R^2^ = 0.999) with applied pressure across all animals (Fig. 2C). Across all conditions, *in situ* PNP values consistently deviated from the identity line and were significantly lower than the corresponding applied pressures in water, demonstrating a systematic pressure reduction upon transcranial transmission (P = 0.002). The difference between the expected applied PNP in water and the estimated *in situ* PNP corresponded to a mean pressure change of −0.33 ± 0.87 dB across the six skulls.

To define the relationship between acoustic pressure and the spatial extent of BBB/BTB opening, beam width and height were evaluated across a range of applied PNPs and pressure thresholds (Fig. 2D, Fig. S7). Excluding threshold levels dominated by zero responses, inter-replicate variability across all pressure groups had a mean coefficient of variation of ∼12%, with an interquartile range of 7–16%, supporting skull-induced variability remained relatively constant across animals and pressure levels. Beam width was defined as the largest lateral extent of the three-dimensional intracranial region exceeding a specified pressure threshold, while beam height was defined as the corresponding axial extent. Rather than assuming a fixed BBB/BTB opening threshold a priori, this analysis was performed to define the acoustic parameter space prior to *in vivo* experimentation, enabling empirical determination of the effective threshold from MRI-based BBB/BTB opening measurements.

At each applied PNP, beam width and height exhibited a strong linear dependence on the selected pressure threshold, with all linear fits yielding R^2^ > 0.97 and p < 0.005 across animals (Fig. 2D, Fig. S7). Because the simulated beam height could exceed the anatomical height of the mouse brain, beam width was used for experimental analyses. For an applied PNP of 0.45 MPa, beam width decreased linearly with a slope of −11.9 mm/MPa. At a lower applied PNP of 0.25 MPa, beam width decreased with a steeper slope of −20.0 mm/MPa.

### 2.3 Validation studies in healthy mice

**Fig. 3:**
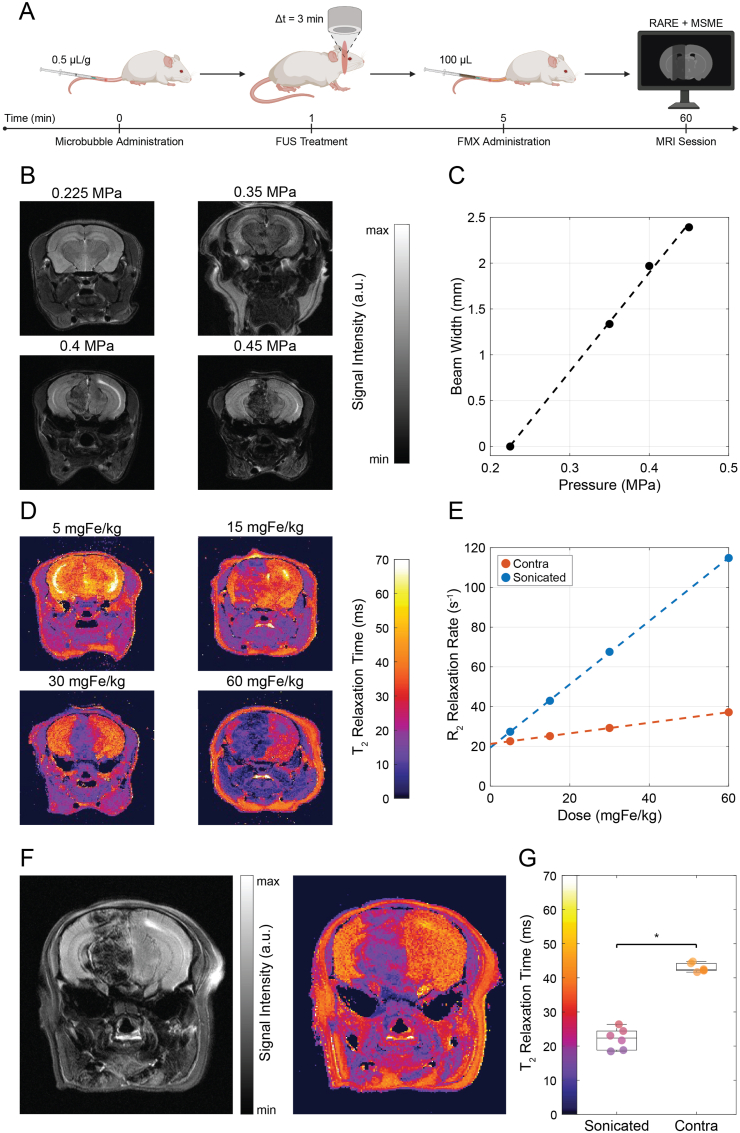
*In Vivo* Studies on Healthy Mice. (**A**) Schematic timeline of the *in vivo* experimental workflow, showing the procedures performed on healthy mice at each time point. (**B**) Representative T_2_-weighted magnetic resonance (MR) images of the brain at varying *in situ* focused ultrasound (FUS) pressures, and after administration of ferumoxytol (FMX, 30 mgFe/kg), showing pressure-dependent widening of the sonicated region. (**C**) Correlation scatter plot between beam width and *in situ* FUS pressure, showing a linear dependence (black dashed line, R^2^ = 0.998, P = 0.001, N = 4). (**D**) T_2_ maps of the brain at varying administered FMX doses after ultrasound treatment (0.45 MPa), showing dose-dependent shortening of T_2_ relaxation time. (**E**) Correlation scatter plots between transverse relaxation rate and administered dose in sonicated (0.45 MPa) and contralateral areas, showing linear dependence in both sonicated (blue dashed line, R^2^ = 0.999, P < 0.001) and contralateral (orange dashed line, R^2^ = 0.999, P < 0.001) areas (N = 4). (**F**) T_2_-weighted brain MR image and corresponding T_2_ map of a healthy mouse after ultrasound treatment (0.45 MPa) and FMX administration (30 mgFe/kg). (**G**) Scatter-box plot comparing T_2_ relaxation times in the sonicated and contralateral brain regions following FUS treatment and FMX administration, highlighting a significant statistical difference (P = 0.016, N = 6). (For interpretation of the references to color in this figure legend, the reader is referred to the Web version of this article.)

To validate microbubble-mediated pressure-dependent BBB opening and FMX dose-dependent accumulation, we performed MRI studies in healthy mice (Fig. 3A).

Analyses of administered microbubble stability showed no significant time-dependent changes in size or concentration (Fig. S8–S10, cf. Supplementary Text) [29].

T_2_-weighted brain MR images acquired at increasing *in situ* FUS pressures showed a pressure-dependent widening of the sonicated region (Fig. 3B). Quantification of beam width revealed a linear relationship with *in situ* FUS peak negative pressure (R^2^ = 0.998, P = 0.001, N = 4; Fig. 3C).

To examine the effect of nanoparticle dose on MRI contrast, mice were treated with microbubbles and FUS at 0.45 MPa and injected with increasing doses of FMX. T_2_ maps showed progressive shortening of T_2_ relaxation time with increasing FMX dose in the sonicated brain region (Fig. 3D). Linear correlations were observed between transverse relaxation rate and FMX dose in both the sonicated region (R^2^ = 0.999, P < 0.001, N = 4) and the contralateral, non-sonicated hemisphere (R^2^ = 0.999, P < 0.001, N = 4) (Fig. 3E). The slope difference was statistically significant (P < 0.001), indicating a significantly higher FMX-dependent relaxivity in the sonicated tissue.

Representative T_2_-weighted images and corresponding T_2_ maps acquired after FUS treatment (0.45 MPa) and FMX administration (30 mgFe/kg) demonstrated a localized reduction in T_2_ relaxation times within the sonicated brain region compared with the contralateral side (Fig. 3F). Quantitative analysis of regional T_2_ relaxation time values showed a statistically significant difference between sonicated and contralateral hemispheres (P = 0.016, N = 6) (Fig. 3G). The T_2_ relaxation time values were 22.36 ms (IQR 5.61 ms) in the sonicated region and 42.38 ms (IQR 2.13 ms) in the contralateral region.

Longitudinal T_2_ maps were acquired to follow FMX clearance over time in healthy mice after microbubble-mediated FUS treatment (Fig. S11), demonstrating progressive significant recovery of T_2_ relaxation times in the sonicated region (P < 0.001), consistent with FMX washout. Quantitative analysis showed that FMX clearance followed an exponential recovery within the first 24 h (R^2^ = 0.990), indicating that the nanoparticle-associated T_2_ effects were restored during the early post-administration period. Fluoro-Jade C staining performed on brain tissue slides at 24 h showed no detectable positive neurons in the sonicated brain and no significant difference in fluorescence intensity between the sonicated and contralateral hemispheres (Fig. S12).

FUS-mediated BBB opening at 650 kHz was validated using the clinical molecular MRI probe gadobutrol (GDB; 0.3 mmol/kg), which produced localized T_1_-weighted contrast enhancement in healthy brain tissue (Fig. S13, cf. Supplementary Text).

**Fig. 4:**
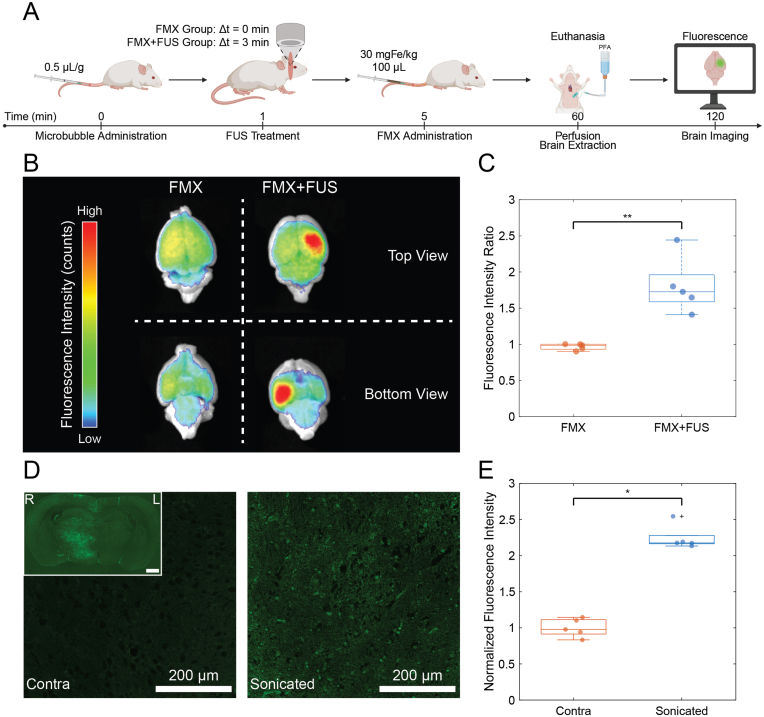
Fluorescence Studies on Healthy Mice. (**A**) Schematic timeline of the experimental workflow for the fluorescence postmortem study, showing the procedures performed on healthy mice at each time point. (**B**) Representative fluorescence images of excised mouse brains with and without microbubble-mediated focused ultrasound (FUS) treatment, showing increased fluorescence in the treated hemisphere. (**C**) Scatter-box plot comparing the fluorescence intensity ratios (sonicated *vs.* contra) between treatment group (FMX + FUS) and control group (FMX), highlighting a significant statistical difference (P = 0.008, N = 5). (**D**) Representative fluorescence images of brain histology slides of mice treated with microbubble-mediated focused ultrasound (FUS), showing increased fluorescence intensity in the sonicated area. In the inset, a full-slide fluorescence scan (scale bar, 1 mm). (**E**) Scatter-box plot comparing the normalized fluorescence intensity between sonicated and contralateral areas of mouse brains treated with FMX + FUS, highlighting a significant statistical difference (P = 0.031, N = 5).

Mice were treated with FMX-FITC with or without FUS and sacrificed for whole-brain and histological fluorescence imaging (Fig. 4A). Representative fluorescence images of excised brains showed increased fluorescence intensity in the hemisphere exposed to FUS compared with the untreated contralateral side (Fig. 4B). Quantification of fluorescence intensity ratios (sonicated vs. contralateral) demonstrated a significant increase in the FMX + FUS group compared with FMX alone (P = 0.008, N = 5; Fig. 4C). The fluorescence ratios were 0.985 (IQR 0.069) for FMX and 1.726 (IQR 0.373) for FMX + FUS.

Fluorescence microscopy of brain sections confirmed higher fluorescence intensity within the sonicated region (Fig. 4D), consistent with the whole-brain imaging. Quantitative analysis of normalized fluorescence intensity showed a significant difference between sonicated and contralateral regions in FMX + FUS-treated mice (P = 0.031, N = 5; Fig. 4E), with values of 2.176 (IQR 0.115) and 0.978 (IQR 0.198), respectively.

### 2.4 FUS-mediated ferumoxytol delivery to glioblastoma

**Fig. 5:**
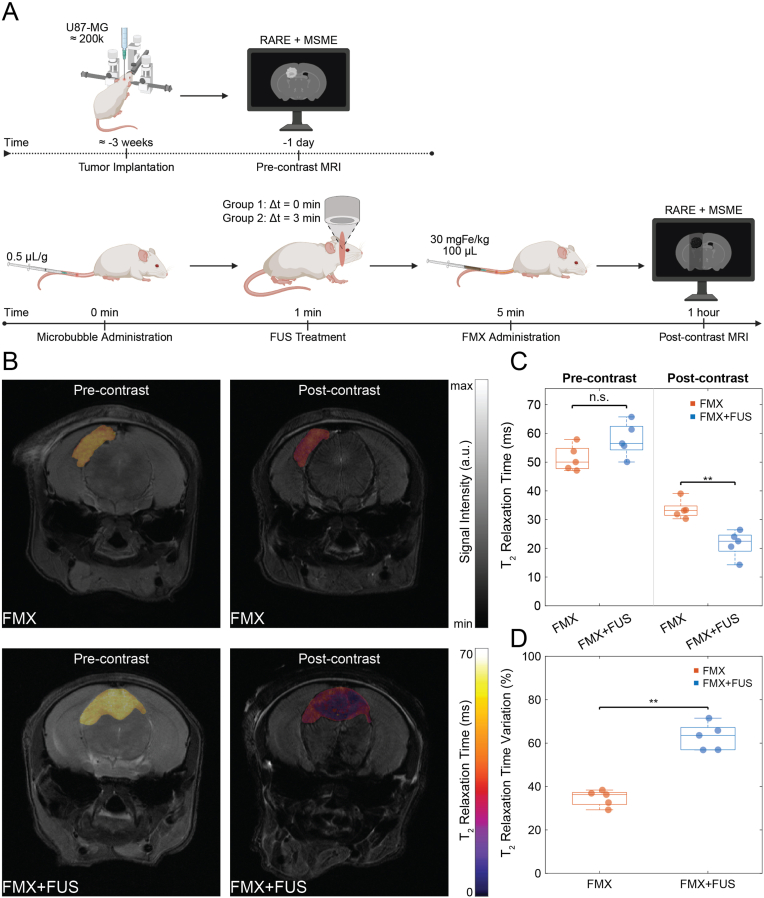
*In Vivo* Studies on Tumor-bearing Mice. (A) Schematic timeline of the *in vivo* experimental workflow, showing the procedures performed on xenografted mice at each time point. (**B**) Representative pre-contrast and post-contrast T_2_-weighted brain MR images of tumor-bearing mice with T_2_ map overlaid on tumor area (Group 1: FMX; Group 2: FMX + FUS). (**C**) Scatter-box plot comparing the pre-contrast and post-contrast T_2_ relaxation times between treatment group (FMX + FUS) and control group (FMX), highlighting a non-significant difference in pre-contrast MRI (P = 0.095, N = 5) and a significant statistical difference in post-contrast MRI (P = 0.008, N = 5). (**D**) Scatter-box plot comparing the T_2_ relaxation time percentage variation between treatment group (FMX + FUS) and control group (FMX), highlighting a significant statistical difference (P = 0.008, N = 5).

To evaluate the impact of BTB opening on FMX delivery to brain tumors, we performed MRI scans before and after FMX administration in NSG mice with implanted U87 glioblastoma cells (2 × 10^5^), comparing microbubble-mediated FUS-treated subjects against sham-treated controls (Fig. 5A).

Tumors were localized and segmented on coronal T_2_-weighted MR images using eye centers as anatomical reference points (Fig. S14A). Three-dimensional reconstruction enabled estimation of tumor position relative to the midpoint between the eyes and calculation of tumor barycenters (Fig. S14B). Baseline tumor volumes were not significantly different between FMX and FMX + FUS groups (P = 1, N = 5; Fig. S14C), with volumes of 11.72 mm^3^ (IQR 1.77 mm^3^) and 11.66 mm^3^ (IQR 4.30 mm^3^), respectively. Baseline (pre-contrast) tumor T_2_ relaxation time values were also not significantly different between FMX [50.03 ms (IQR 7.07 ms)] and FMX + FUS [56.51 ms (IQR 8.20 ms)] groups (P = 0.095, N = 5; Fig. 5B–C).

After FMX administration, all tumors demonstrated a hypointense (dark) signal enhancement at 1 h (Fig. 5B). The T_2_ relaxation time of FUS-treated tumors [22.47 ms (IQR 5.62 ms)] was significantly lower compared with untreated control tumors [33.14 ms (IQR 3.26 ms); P = 0.008, N = 5; Fig. 5C]. Tumor T_2_ relaxation time variation was 36.28% (IQR 5.50%) in the FMX group and 63.58% (IQR 10.33%) in the FMX + FUS group, with a significant difference between groups (P = 0.008, N = 5; Fig. 5D). Based on the median pre- and post-contrast tumor T_2_ values, tumor transverse relaxation rate change (ΔR_2_) increased from 10.19 s^−1^ in the FMX group to 26.81 s^−1^ in the FMX + FUS group, corresponding to a 2.6-fold enhancement under FUS conditions.

**Fig. 6:**
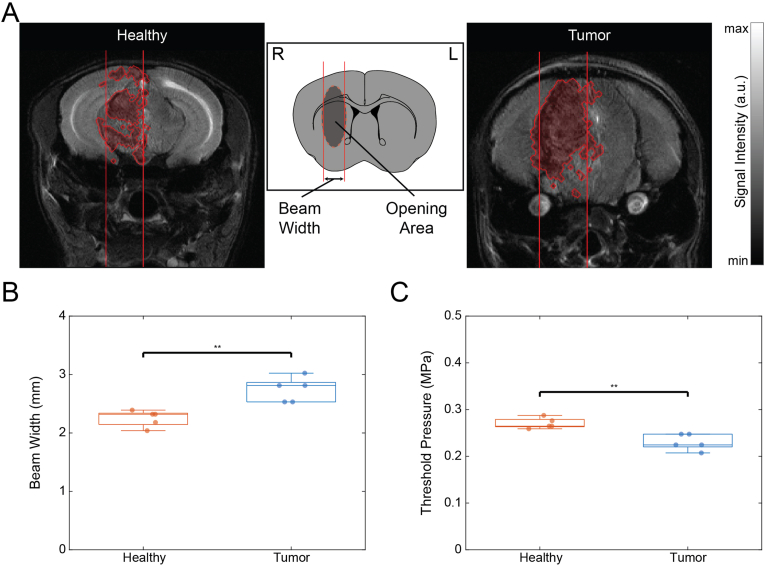
Microbubble-mediated FUS Threshold for Nanoparticle Delivery. (**A**) Post-contrast T_2_-weighted MRI of a healthy (left) and a tumor-bearing (right) mouse after focused ultrasound (FUS) treatment and FMX administration. Significant BBB/BTB opening is shown in red with vertical lines representing the beam width. (**B**) Scatter-box plot of the quantified contrast-enhanced beam width in healthy and tumor tissues (N = 5 per group), showing a significant difference between groups (P = 0.008). (**C**) Scatter-box plot of the estimated BBB/BTB opening threshold pressure in healthy and tumor tissues (N = 5 per group), showing a significant difference between groups (P = 0.008). (For interpretation of the references to color in this figure legend, the reader is referred to the Web version of this article.)

Using the validated pressure of 0.45 MPa, the width of significant FMX accumulation differed between healthy and tumor tissue (Fig. 6A, Fig. S15). Quantification showed a significantly increased beam width of 2.81 mm (IQR 0.33 mm) in tumor tissue compared with 2.32 mm (IQR 0.19 mm) in healthy tissue (Fig. 6B). Using the expected beam width found in simulations (Fig. 2D), the pressure threshold for significant FMX accumulation was significantly lower in the tumor [0.224 MPa (IQR 0.027 MPa)] relative to healthy tissue [0.265 MPa (IQR 0.016 MPa); P = 0.008; Fig. 6C].

**Fig. 7:**
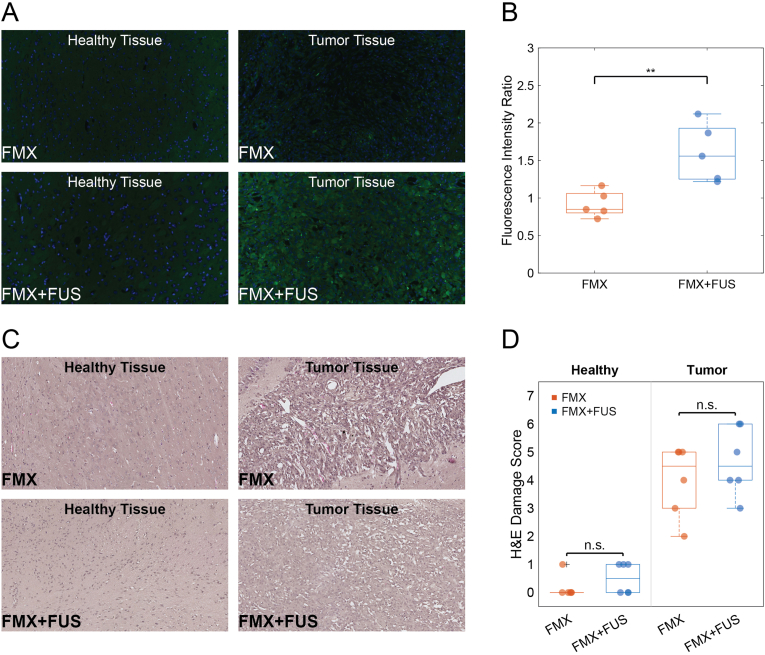
Histological Analysis. (**A**) Representative fluorescence images of brain histology slides of xenografted mice from Group 1 (FMX) and Group 2 (FMX + FUS), showing a higher fluorescence intensity in the tumor area of FMX + FUS mice. (**B**) Scatter-box plot comparing the green fluorescence intensity ratios of mice from groups FMX and FMX + FUS, highlighting a significant statistical difference (P = 0.008). (**C**) Hematoxylin and Eosin (H&E) stained slides of brain tissue (healthy and tumor) from mice of groups FMX and FMX + FUS. (**D**) Scatter-box plot comparing the estimated H&E damage score of mice from groups FMX and FMX + FUS, showing a non-significant difference in both healthy (P = 0.545) and tumor tissues (P = 0.472). (For interpretation of the references to color in this figure legend, the reader is referred to the Web version of this article.)

*Postmortem* fluorescence imaging of brain tissue sections showed higher fluorescence intensity within tumor regions of FUS-treated tumors compared with untreated controls (Fig. 7A). Quantitative analysis demonstrated significantly higher tumor-to-healthy-tissue fluorescence intensity ratios in FUS-treated tumors [1.558 (IQR 0.679)] compared with untreated controls [0.849 (IQR 0.259); P = 0.008; Fig. 7B], corresponding to a 1.8-fold increase in the tumor-to-healthy-tissue fluorescence ratio under FUS conditions. Tumor tissue showed a significantly higher cell density than healthy brain (5513 nuclei/mm^2^, IQR = 613 vs 1950 nuclei/mm^2^, IQR = 153; P = 0.002; Fig. S16).

Histopathological evaluation of H&E-stained sections from both healthy brain and tumor tissue showed no qualitative differences between treatment groups (Fig. 7C). Accordingly, semi-quantitative scores for tissue damage revealed no significant difference between FMX and FMX + FUS in either healthy tissue (P = 0.545) or tumor tissue (P = 0.472; Fig. 7D), with median damage scores of 0.0 (IQR 0.0) vs. 0.5 (IQR 1.0) in healthy brain and 4.5 (IQR 2.0) vs. 4.5 (IQR 2.0) in tumor.

## 3 Discussion and conclusion

Our results showed that microbubble-mediated FUS treatment at a clinically relevant frequency enhanced nanoparticle delivery to glioblastomas, producing a localized and quantitatively measurable increase in intratumoral FMX accumulation, as detected by MRI and confirmed by fluorescence imaging. When extended to individualized mouse skull geometries, simulations at 650 kHz revealed pronounced standing wave formation within the cranial cavity, resulting in spatially heterogeneous pressure modulation. Peak-to-mean pressure ratios were consistently elevated across animals with modest inter-animal variability, indicating reproducible interference-driven amplification despite differences in skull anatomy. These simulation results are consistent with *in vivo* observations in rats by O'Reilly et al. where FUS combined with microbubbles at 0.25-1 MHz produced spatially localized BBB opening/disruption and heterogeneous contrast enhancement in sonicated regions, reflecting non-uniform acoustic pressure distributions across the skull [28]. In our context, these standing waves contributed substantially to pressure modulation within the brain and accounted for nearly the full pressure attenuation observed across the skull relative to water-only conditions [28], thus providing a mechanistic explanation for the relatively small net pressure changes observed following transcranial transmission.

While standing wave formation was prominent in the mouse brain at 650 kHz due to the small cranial dimensions and strong boundary reflections, these effects are expected to be substantially reduced in the human brain. At clinical scales, the larger cranial cavity relative to the acoustic wavelength shifts resonance conditions and diminishes constructive interference within the parenchyma, resulting in smoother pressure distributions. Prior clinical and preclinical studies in large animals and humans have reported reduced standing wave contributions at comparable or lower frequencies [30,31]. The standing-wave associated heterogeneity observed in our study represents a conservative modeling scenario and underscores the importance of subject-specific simulations when translating ultrasound parameters across species.

Across applied peak negative pressures ranging from 0.25 to 0.45 MPa, estimated *in situ* peak negative pressures scaled linearly with applied pressure in water. Across animals, transcranial transmission resulted in a mean pressure reduction of −0.33 ± 0.87 dB relative to water, indicating modest but variable attenuation across skulls. Li et al. showed similar values at 500 kHz (−0.92 dB) [16]. *In situ* pressures consistently deviated from the identity line, confirming systematic pressure reduction across the skull; however, the magnitude of this reduction was smaller than predicted by bulk skull attenuation models, consistent with partial pressure recovery arising from intracranial reflections and interference effects. Together, these findings highlight the importance of accounting for intracranial interference effects, rather than assuming homogeneous attenuation, when translating applied pressures to *in situ* exposure.

Pagé et al. reported that ∼79% of FUS-mediated delivery studies rely primarily on indirect T_1_-weighted contrast-enhanced MRI with gadolinium to assess BBB opening [32]; in contrast, our approach employs FMX as an intrinsically quantifiable iron-oxide nanoparticle and uses T_2_-based relaxometry to directly map nanoparticle accumulation. T_2_-weighted MRI demonstrated that FUS enables spatially controlled modulation of FMX accumulation in the healthy mouse brain. The linear dependence of beam width on *in situ* FUS pressure indicates that the extent of the affected brain volume can be predictably tuned. Furthermore, the linear relationship between FMX dose and transverse relaxation rate confirms that FMX provides quantitative MRI contrast *in vivo* in both sonicated and non-sonicated tissue. Consistent with these relationships, the significant reduction in the T_2_ relaxation time observed in the sonicated hemisphere following FUS and FMX administration indicates localized enhancement of FMX accumulation in the treated region. Payne et al. proposed a dual-mode imaging framework for microbubble-mediated FUS delivery of liposomes across the BBB, using Gd-loaded liposomes for quantitative T_1_-weighted MRI together with near-infrared fluorescence labeling for *ex vivo* validation [33]; in our work, FMX was covalently conjugated with FITC to enable direct visualization of probe delivery and to corroborate quantitative MRI findings with *ex vivo* optical readouts. MRI and histological fluorescence showed concordant enhancement of FMX accumulation under FUS conditions. Relative to FMX alone, the FMX + FUS group showed lower post-contrast tumor T_2_ relaxation times, a 2.6-fold increase in ΔR_2_ by MRI, and a 1.8-fold increase in the tumor-to-healthy-tissue fluorescence ratio by histological fluorescence imaging, supporting the same conclusion of enhanced probe accumulation after FUS. Importantly, FITC conjugation also serves as a representative chemical functionalization strategy for FMX, which in future studies could be adapted to attach or co-formulate therapeutic payloads for FMX-based theranostic designs [23,25].

While several groups have investigated microbubble-mediated FUS-enhanced delivery of small molecules [34], antibodies [35,36], or liposomal carriers [37] in glioblastoma models, recent studies have further advanced FUS-mediated delivery across the BBB using clinically relevant low-frequency ultrasound regimes. Shumer-Elbaz et al. demonstrated low-frequency FUS-enabled delivery of lipid nanoparticle RNA formulations to glioblastoma [38], and Todd et al. compared acute gadolinium enhancement with later AAV9-GFP distribution after FUS-mediated BBB opening [39]. Our work demonstrates microbubble-mediated FUS enhancement of an iron-oxide nanoparticle that is both clinically translatable and directly quantifiable by MRI relaxometry, providing delivery pressure thresholds in tumor versus healthy brain tissue and offering a quantitative basis for selective nanoparticle delivery in future translational studies. Notably, the 2.6-fold increase in tumor transverse relaxation rate change under FUS conditions provides a quantitative estimate of enhanced FMX delivery efficiency in the tumor model. Inter-animal variation in tumor position was observed, consistent with biological variability in the orthotopic model, addressed by individualized MRI-guided FUS targeting. The absence of increased tissue damage on H&E staining indicates that this enhanced delivery was achieved without detectable structural injury to either tumor or surrounding brain tissue. The lower acoustic pressure threshold required to induce nanoparticle accumulation in tumor compared with healthy brain is consistent with the structurally and functionally abnormal vasculature and barrier properties of glioblastoma [[40], [41], [42], [43]]. This differential threshold finding aligns with reports of tumor vasculature susceptibility to mechanical modulation [44,45] but, to our knowledge, provides one of the first direct comparisons of threshold pressures for nanoparticle delivery between healthy and tumor tissues in the context of a clinically relevant FUS frequency regime and a nanoparticulate iron-oxide agent. Tumor vessels exhibit irregular architecture, reduced pericyte coverage, compromised basement membranes, and increased baseline permeability, all of which are expected to lower the mechanical energy required to transiently enhance transport across the vascular barrier [46]. These findings suggest that FUS parameters optimized for tumor delivery may fall below thresholds associated with effects in healthy brain, providing an opportunity to exploit intrinsic tumor vulnerabilities to improve delivery specificity and establish thresholds for tumor-selective nanoparticle transport.

Our work employed components with established clinical precedents. FMX has recently been FDA-approved as a contrast agent for imaging brain cancer [22] and microbubble-mediated BBB opening/disruption with FUS is actively being evaluated in multiple clinical trials for brain cancer [[47], [48], [49]]. The use of a clinically relevant FUS frequency together with FMX, an FDA-approved, intrinsically MRI-detectable, and chemically functionalizable nanoprobe, strengthens the translational relevance of this work and provides a quantitative proof-of-concept platform for enhancing nanoparticle delivery to brain tumors. Its combination with microbubble-mediated FUS may improve intratumoral delivery of theranostic iron-oxide formulations. In the future, this achievement can enable sufficient drug delivery at lower administered doses, reducing off-target sequestration in liver and spleen and improving the safety profile of FMX-based nanomedicines. Although this study focused on the delivery of FMX in a representative xenograft model, the same approach can be extended to investigate longitudinal nanoparticle retention, spatial heterogeneity within tumors, and correlations with therapeutic response. This framework may also enable future studies with different nanoparticle formulations to determine how particle size, composition, and surface properties affect FUS-mediated delivery across the BBB. Overall, our results establish a pathway toward image-guided, dose-efficient nanoparticle delivery strategies aimed at improving treatment outcomes for patients with glioblastoma.

## 4 Materials and methods

### 4.1 Materials

Ferumoxytol (FMX; Feraheme, 30 mgFe mL^−1^) was obtained from AMAG Pharmaceuticals. Gadobutrol (GDB; Gadavist®, 1.0 mmol/mL) was obtained from Bayer Healthcare Pharmaceuticals. Epichlorohydrin (ECH, ≥99%), sodium hydroxide (NaOH, ≥98%), ammonium hydroxide solution (NH_4_OH, 28–30%), sodium bicarbonate (NaHCO_3_, ≥99.5%), anhydrous sodium carbonate (Na_2_CO_3_, ≥99.5%), fluorescein isothiocyanate (FITC, isomer I), dimethyl sulfoxide (DMSO, anhydrous, ≥99.9%), and nitric acid (HNO_3_, ≥69%), phosphate-buffered saline (PBS, pH 7.4), fetal bovine serum (FBS), sterile saline (0.9% NaCl), and Trypsin–EDTA (0.25%) were purchased from Fisher Scientific. UltraPure™ Agarose and Dulbecco's Modified Eagle Medium (DMEM) were obtained from Invitrogen. 4′,6-diamidino-2-phenylindole (DAPI) and Fluoro-Jade® C RTD™ Stain Reagent Kit were purchased from VWR. Dialysis tubing (molecular weight cutoff 12–14 kDa) and centrifugal ultrafiltration units (10 kDa cutoff) were obtained from Spectrum Laboratories and Millipore, respectively. Definity microbubbles (perflutren lipid microspheres) were purchased from Lantheus Medical Imaging. The human glioblastoma cell line U87-MG was obtained from the American Type Culture Collection (ATCC).

### 4.2 Ferumoxytol conjugation

FMX (2.5 mL, 30 mg mL^−1^ Fe) was diluted with 4 mL Milli-Q water and mixed with 10 mL of 5 M NaOH under gentle stirring at room temperature. ECH (4 mL) was added, and the reaction was allowed to proceed for 24 h to generate epoxide groups on the FMX surface. Excess ECH and low-molecular-weight byproducts were removed by dialysis (12-14 kDa molecular weight cutoff) against Milli-Q water for 3 days with frequent water changes.

The epoxide-activated FMX was reacted with NH_4_OH (10 mL) at 37 °C for 24 h under continuous stirring, promoting nucleophilic epoxide ring opening and formation of surface amines. The resulting amine-functionalized FMX was purified by a second dialysis step (12-14 kDa cutoff) against Milli-Q water for 3 days and stored in aqueous suspension.

FITC was conjugated to amine-functionalized FMX under mildly alkaline conditions using carbonate-bicarbonate buffer (0.1 M, pH 9.0). Amine-functionalized FMX (6 mL) was buffer-exchanged into carbonate–bicarbonate buffer and concentrated to 5 mL using 10 kDa centrifugal ultrafiltration units.

FITC was freshly dissolved in anhydrous DMSO at 1 mg mL^−1^, and 1 mL of this solution was added slowly to the nanoparticle suspension in 50 μL aliquots under continuous stirring. The reaction mixture was incubated for 8 h at 4 °C in the dark to minimize photobleaching and non-specific dye degradation.

Unreacted FITC was quenched by addition of NH_4_OH to a final concentration of approximately 50 mM, followed by incubation for 2 h at 4 °C in the dark. The resulting FMX-FITC nanoparticles were purified by repeated washing with carbonate-bicarbonate buffer using centrifugal filtration and further dialysis (12-14 kDa cutoff) against Milli-Q water for 2 days to remove unbound FITC and residual small-molecule reagents. Purified FMX-FITC was stored at 4 °C protected from light until use.

### 4.3 Ferumoxytol characterization

Iron content of the nanoparticle formulations was quantified by inductively coupled plasma optical emission spectroscopy (ICP-OES) using an iCAP 6300 (Thermo Fisher Scientific, USA) after acid digestion of the samples in nitric acid. Hydrodynamic size and size distribution of the nanoparticles were measured by dynamic light scattering (DLS) using a Brookhaven Instruments NanoBrook Omni operated at 25 °C with a scattering angle of 90°. Samples were diluted in Milli-Q water to a final iron concentration of 50 μg mL^−1^ prior to measurement. Data were analyzed using MATLAB, and results are reported as intensity-weighted size distributions with corresponding polydispersity indices. Transmission electron microscopy (TEM) was used to determine the dry particle size and morphology. Samples were deposited on copper grids and imaged using a Tecnai G2 F20 X-TWIN (FEI, USA) operating at 200 kV. Dry particle diameters were quantified by manually measuring nanoparticle sizes using ImageJ (N = 200), and size distributions were calculated from these measurements. UV-visible absorption spectra were acquired using an Agilent Cary 60 UV-vis spectrophotometer (Agilent Technologies, USA) over the wavelength range 420-580 nm with an averaging time of 0.1 s, data interval of 0.5 nm, and a scan rate of 300 nm min^−1^. Nanoparticle dispersions were diluted in PBS, background absorbance from PBS was subtracted, and spectra were normalized at 420 nm prior to analysis. The spectra were smoothed by averaging every 8 consecutive data points, and the difference between the normalized FMX-FITC and FMX spectra was calculated to isolate the FITC-specific absorbance contribution. Fluorescence excitation–emission matrices of FMX-FITC were acquired using a Tecan Infinite M1000 multimode microplate reader (Tecan Group Ltd., Männedorf, Switzerland) in top-read fluorescence mode. Emission spectra were recorded from 400 to 800 nm with a step size of 5 nm, while excitation wavelengths were scanned from 300 to 700 nm with a step size of 5 nm. The emission bandwidth was set to 5 nm. The detector gain was set to 100 (manual), with 50 flashes per data point at a flash frequency of 400 Hz, an integration time of 20 μs, and zero lag and settle times. The Z-position was fixed at 20,000 μm. Prior to normalization, the excitation-emission identity line ±10 nm was removed by spline interpolation to suppress elastic scattering and residual laser contributions. The data were then normalized to 100% of the global maximum of the excitation-emission matrix. One-dimensional excitation and emission spectra were extracted at λ_em_ = 520 nm and λ_ex_ = 495 nm, respectively. Transverse relaxivity (r_2_) measurements were performed using agar phantoms (3% w/v) prepared from UltraPure™ Agarose (Invitrogen) in Milli-Q water. Agarose was dissolved at 70 °C, after which FMX or FMX-FITC was added to achieve final iron concentrations of 0, 0.0625, 0.125, 0.25, 0.5, and 1 mM. The mixtures were immediately transferred into 2 mL vials and rapidly cooled to allow gelation. Phantoms were imaged on a 7 T MRI scanner (Bruker BioSpin, Billerica, MA) using a T_2_-weighted multi-slice multi-echo (MSME) sequence with TR = 2200 ms, TE = 7.5 to 105 ms in 7.5 ms increments, field of view = 18.0 × 18.0 mm, matrix = 192 × 192, slice thickness = 0.7 mm, and interslice spacing = 1.0 mm. T_2_ maps were generated in MATLAB, and r_2_ was obtained from the linear fit of 1/T_2_ versus iron concentration.

### 4.4 Ultrasound simulations

A set of 6 male C57BL/6 mice was imaged on a Quantum GX micro-CT (Waltham, Massachusetts, USA) to simulate the field during FUS treatment. The obtained micro-CT image was 474×504×921 with a cubic voxel size of 0.049 mm. The images were resampled linearly to a cubic voxel size of 0.2 mm. The bone, soft tissue, and water were isolated based on their Hounsfield units (300 HU for bone/soft tissue and −70 HU for soft tissue/water threshold). Density and sound speed were linearly interpolated in each region using hounsfield2density, a predefined function in the k-Wave MATLAB (Natick, Massachusetts, USA) toolbox [50]. The region surrounding the animal was defined as water. The transducer was described as a bowl with a diameter and radius of curvature of 40 mm. At a center frequency of 0.65 MHz, the simulations used 12 points per wavelength in water and a Courant-Friedrichs-Lewy stability criterion of 0.1, leading to a time step of 22.8 ns. The simulation was run for 67 μs, allowing the initial wave to travel to the length of the simulation grid and back past the top of the skull (100 mm). Simulations were conducted using the k-Wave toolbox [50].

### 4.5 Animal experiments

All animal procedures were performed in accordance with the guidelines of the Stanford University Administrative Panel on Laboratory Animal Care (APLAC) and approved under protocol 12040. CD-1 mice (Crl:CD1(ICR); Charles River Laboratories) were used for experiments in healthy brain tissue to study and optimize nanoparticle delivery under focused ultrasound. Female NSG mice (NOD.Cg-Prkdc^scid^ Il2rg^tm1Wjl^/SzJ; Jackson Laboratory, catalog no. 005557) were used for intracranial tumor implantation experiments.

### 4.6 Orthotopic xenograft model

Orthotopic brain tumors were established in NSG mice by stereotactic intracranial implantation of U87 glioblastoma cells (passages 10-12) under inhalation anesthesia. Anesthesia was induced with 4-5% isoflurane in oxygen (2 L min^−1^) and maintained at 1-3% isoflurane via a nose cone. Body temperature was maintained at 37 °C using a warming pad, and depth of anesthesia was monitored by respiratory rate and toe-pinch reflex.

Preoperative analgesia was administered subcutaneously with buprenorphine SR (0.5-1 mg kg^−1^). Local anesthesia (bupivacaine, 1-2 mg kg^−1^ SQ) was applied at the incision site. Carprofen (10-25 mg kg^−1^ SQ) was administered postoperatively and repeated every 24 h for up to 2 days as needed. All surgical procedures were performed under aseptic conditions using sterilized instruments and drapes.

Mice were placed in a stereotactic frame, and the scalp was disinfected and incised (∼5 mm). A burr hole (∼0.2 mm) was drilled at coordinates 0.5 mm lateral (right) and 0.7 mm anterior to lambda. A suspension of 2 × 10^5^ U87 cells in 4 μL HBSS was injected at a depth of 2 mm. After injection, the needle was left in place for at least 2 min and then withdrawn slowly (∼0.5 mm min^−1^) to prevent reflux. The burr hole was sealed with bone wax, and the skin was closed with dermal glue or surgical clips.

Following surgery, mice were kept on warming pads until fully recovered and were monitored for respiration, movement, and general activity. Animals were checked daily for general health, activity, and body weight.

### 4.7 *In vivo* magnetic resonance imaging

Mice were anesthetized and ophthalmic ointment was applied to both eyes to prevent corneal drying. Animals were positioned supine on an MRI-compatible temperature probe, and body temperature was maintained using a warm-air blower and heating system throughout imaging. Anesthesia depth was confirmed by the absence of a toe-pinch reflex and a stable respiratory rate prior to scan initiation. Respiration was continuously monitored, and total scan time did not exceed 60 min. MRI experiments were performed on the 7 T MRI scanner (Bruker BioSpin, Billerica, MA). Brain imaging included T_2_-weighted MRI and multi-echo T_2_ mapping. T_2_-weighted images were acquired using a 2D TurboRARE (Bruker RARE) sequence with TR = 2500 ms, effective TE = 33 ms, field of view = 18 × 18 mm, matrix = 256 × 256, slice thickness = 0.7 mm, interslice spacing = 1.0 mm, echo train length = 8, and number of averages = 6. For T_2_ mapping, we employed a multi-slice multi-echo (MSME) sequence with TR = 2200 ms, TE = 7.5 to 105 ms in 7.5 ms increments, field of view = 18.0 × 18.0 mm, matrix = 192 × 192, slice thickness = 0.7 mm, and interslice spacing = 1.0 mm. In mice administered GDB, T_1_-weighted images were acquired using a T_1_ RARE sequence with TR = 750 ms, TE = 6.5 ms, field of view = 18 × 18 mm, matrix = 256 × 256, slice thickness = 0.7 mm, interslice spacing = 1.0 mm, echo spacing = 6.5 ms, RARE factor = 2, and number of averages = 4. T_2_ maps were then generated by voxel-wise mono-exponential fitting of the signal decay using MATLAB. Regions of interest were defined in the sonicated and contralateral hemispheres in healthy mice and in the tumor region and contralateral hemisphere in tumor-bearing mice for quantitative analysis of T_2_ and ΔR_2_. After imaging, mice were transferred to a warmed recovery cage and monitored every 15 min until fully awake and ambulatory.

### 4.8 Tumor localization and size estimation

T_2_-weighted MR images were imported in MATLAB. The right and left eye centers were defined by manually drawing circular ROIs on the corresponding slices. ROI centers were converted from pixels to mm coordinates, yielding two 3D landmarks; the eye midpoint was computed as the vector average of the two eye-center coordinates. Tumors were segmented by manually drawing polygon ROIs on all slices containing tumor. Slice-wise masks were merged by union and reconstructed into a 3D tumor volume by spline interpolation of signed distance fields along Z. Tumor volume was calculated from the voxel count and voxel volume and reported in mm^3^. The tumor barycenter was computed as the centroid of the 3D tumor mask, and tumor location was expressed relative to the eye midpoint.

### 4.9 Focused ultrasound treatment

Mice were anesthetized with isoflurane (induction 2.5-5%, maintenance 1-2%) delivered via a nose cone in medical air. Animals were positioned supine in a stereotaxic holder integrated into the focused ultrasound (FUS) system, and body temperature was maintained using a heated water pad. Hair over the skull was trimmed and then removed using a depilatory cream. The scalp was acoustically coupled to the transducer through degassed water and degassed ultrasound gel. FUS was delivered transcranially using a 650 kHz focused ultrasound transducer designed and built in-house (Fig. S17). A single focal target was sonicated per mouse. For healthy-mouse studies, the target was placed in the right hemisphere. For tumor-bearing mice, the target was placed at the tumor barycenter, which was estimated from pre-contrast MRI based on its position relative to the midpoint between the two eyes. Definity microbubbles were administered intravenously via a tail-vein butterfly catheter at a dose of 0.5 μL g^−1^ (cf. Supplementary Text). Ultrasound was applied at a center frequency of 650 kHz with the estimated *in situ* peak negative pressures ranging from 0 to 0.45 MPa for a total sonication duration of 3 min (post microbubble injection). Contrast agents were administered intravenously directly following FUS treatment, and MRI was used to assess nanoparticle accumulation and relaxation effects in the brain. Animals remained under anesthesia throughout the procedure and were continuously monitored for respiration and physiological stability. Body temperature was maintained using a heated water pad, and fluids were provided as needed.

### 4.10 Whole-brain fluorescence imaging

Mice were placed under deep anesthesia (5% isoflurane) and subjected to transcardial perfusion with 30 mL of 4% paraformaldehyde (PFA). Their brains were carefully extracted and imaged *ex vivo* using an IVIS optical imaging system (PerkinElmer, Waltham, MA) to quantify fluorescence from FITC-labeled nanoparticles. Whole brains were placed in the imaging chamber and imaged in fluorescence mode using the FITC filter set (excitation 500 nm, emission 550 nm). Images were acquired with excitation power 5%, exposure time 1 s, binning 2, f-stop 2.0, and field of view 6. For each brain, fluorescence images were acquired from both the dorsal (top) and ventral (bottom) sides. Fluorescence images were exported and analyzed using ImageJ. Fluorescence intensity (counts) was measured from manually drawn ROIs covering the sonicated (right) and contralateral (left) hemispheres. Background signal was measured from a region outside the tissue and subtracted from all measurements. The hemispheric fluorescence ratio was calculated as the background-corrected intensity in the right hemisphere divided by that in the left hemisphere, and the final value was obtained by averaging the ratios from the top and bottom views.

### 4.11 Histological evaluation

Extracted brains were post-fixed in 10% neutral buffered formalin, dehydrated through graded ethanol, embedded in paraffin, and sectioned into 5 μm-thick slices. One set of sections from tumor-bearing mice was stained with hematoxylin and eosin (H&E) and imaged using a digital slide scanner (NanoZoomer 2.0-RS; Hamamatsu Photonics, Shizuoka, Japan). H&E grading was performed using a standardized rubric (Table S1) designed to assess red blood cell (RBC) extravasation/microhemorrhage, vascular and endothelial alterations, parenchymal vacuolation/edema, and neuronal injury. Each part of this rubric has been previously applied to evaluate BBB opening in both tumor and healthy brain tissue [51,52]. Tissue sections were used for the quantification of FITC fluorescence. Fluorescence images were acquired using an optical fluorescence microscope (Keyence BZ-X700 Series, Japan), and green-channel (FITC filter) fluorescence intensity was quantified using ImageJ. Fold enhancement of fluorescence under FUS was calculated as the ratio of the median tumor-to-healthy-tissue fluorescence intensity ratio in the FMX + FUS group to that in the FMX group. DAPI staining was performed to visualize the cell nuclei in healthy and tumor regions, and cell density was estimated by threshold-based segmentation of the nuclei in MATLAB, normalized to the scanned area. Brain tissue sections from healthy mice were stained with Fluoro-Jade C using the Fluoro-Jade® C RTD™ Stain Reagent according to the manufacturer's protocol to assess acute neurodegeneration. Fluorescence images were acquired using an optical fluorescence microscope (Keyence BZ-X700 Series, Japan), and green-fluorescence signal was quantified using ImageJ.

### 4.12 Studies in healthy mice

A series of *in vivo* experiments in CD-1 mice was performed to quantify how FUS pressure and administered nanoparticle dose influence nanoparticle delivery to brain tissue, and to verify the dual-mode MRI-fluorescence imaging capability of FMX-FITC.

To evaluate the relationship between FUS pressure and beam width, four CD-1 mice received an intravenous injection of Definity microbubbles at t = 0 min. FUS sonication was initiated at t = 1 min and applied to a single focal target in the right hemisphere for 3 min, with each mouse receiving a different peak negative pressure (0.225, 0.35, 0.40, or 0.45 MPa). At t = 5 min, FMX-FITC (30 mgFe/kg) diluted in 100 μL saline was administered intravenously. At t = 60 min, animals were transferred to the MRI scanner for acquisition of T_2_-weighted RARE images. Beam width was quantified from T_2_-weighted MRI using FIJI. A region of interest was defined in the contralateral hemisphere to establish baseline signal intensity, and the enhanced region was defined as voxels differing from the control-region mean by more than two standard deviations (Fig. S15). The enhanced area was delineated on all MRI slices, and the total three-dimensional volume was calculated by summing the areas across slices and multiplying slice thickness. Beam width was then defined as the maximum width of this reconstructed 3D volume.

To investigate the effect of administered FMX dose on nanoparticle accumulation, four CD-1 mice received Definity microbubbles at t = 0 min, followed by FUS sonication at t = 1 min at a fixed pressure of 0.45 MPa for 3 min. At t = 5 min, FMX-FITC was injected intravenously at 5, 15, 30, or 60 mgFe/kg, each diluted in 100 μL saline. MRI acquisition using RARE and MSME sequences was performed at t = 60 min to quantify T_2_ relaxation times in sonicated and contralateral brain regions.

To assess the effect of FUS treatment on regional nanoparticle delivery, six CD-1 mice received Definity microbubbles at t = 0 min, followed by FUS sonication at t = 1 min at a fixed pressure of 0.45 MPa for 3 min, targeting the right hemisphere. At t = 5 min, FMX (30 mgFe/kg) diluted in 100 μL saline was administered intravenously. MRI acquisition using a T_2_-weighted MSME sequence was performed at t = 60 min to quantify T_2_ relaxation times in the sonicated and contralateral brain regions. The beam width of the sonicated area was estimated.

FMX clearance over time after FUS-mediated delivery was investigated in five CD-1 mice, which were administered Definity microbubbles at t = 0 min, followed by FUS sonication at t = 1 min at a fixed pressure of 0.35 MPa for 3 min, targeting the right hemisphere. At t = 5 min, FMX (30 mgFe/kg) diluted in 100 μL saline was administered intravenously. Longitudinal MRI was performed using the T_2_-map MSME sequence at 1, 4, 8, and 24 h after FMX administration to quantify T_2_ relaxation times in the sonicated brain region. T_2_ relaxation times were plotted as a function of time and fitted with an exponential recovery model to assess FMX clearance kinetics. Brain histology slides stained with Fluoro-Jade C were scanned and the fluorescence intensity in the sonicated and contralateral hemispheres was quantified and normalized to the contralateral mean value.

To validate the dual-mode (MRI-fluorescence) imaging capability of FMX-FITC, ten CD-1 mice were administered Definity microbubbles and randomly divided into two groups (FMX and FMX + FUS, n = 5 per group). Mice in the FMX + FUS group underwent FUS sonication at 0.45 MPa for 3 min, whereas mice in the FMX group did not receive ultrasound treatment. At t = 5 min, all mice received FMX-FITC (30 mgFe/kg) diluted in 100 μL saline by intravenous injection. At t = 60 min, mice were deeply anesthetized, perfused with 4% PFA, and brains were extracted and post-fixed in PFA for whole-brain fluorescence imaging. Brains were imaged using an IVIS system to quantify hemispheric fluorescence distribution. Brain tissues from the FMX + FUS group were subsequently processed to obtain histology slides for FITC detection with fluorescence microscopy. Fluorescence intensity of sonicated and contralateral areas was estimated and normalized to the contralateral mean value.

To validate FUS-mediated delivery using a small-molecule MRI contrast agent, four healthy CD-1 mice were administered GDB. Mice received Definity microbubbles at t = 0 min, followed by FUS sonication at t = 1 min at a fixed pressure of 0.35 MPa for 3 min, targeting the right hemisphere. At t = 5 min, GDB (0.3 mmol/kg) was administered intravenously. MRI acquisition was performed at t = 15 min using the T_1_-weighted RARE sequence to calculate sonicated beam width and threshold pressure.

### 4.13 Study of nanoparticle delivery to tumors

To evaluate nanoparticle delivery to brain tumors, orthotopic glioblastoma xenografts were established in 10 NSG mice approximately 3 weeks prior to imaging. A pre-contrast MRI session was performed 1 day before treatment (t = −1 day) to determine baseline tumor T_2_ relaxation times, to localize the tumor, and to estimate tumor size and barycenter using the image-based tumor localization workflow described above. At t = 0 min, mice received an intravenous injection of Definity microbubbles and were randomly assigned to two groups (FMX and FMX + FUS, N = 5 per group). At t = 1 min, FUS sonication was applied only in the FMX + FUS group for 3 min, targeting the tumor barycenter calculated from the pre-contrast MRI. At t = 5 min, all mice received FMX-FITC (30 mgFe/kg) diluted in 100 μL saline by intravenous injection. A post-contrast MRI session was performed at t = 60 min using T_2_-weighted RARE and MSME sequences. Tumor T_2_ relaxation times were computed from MSME data, and T_2_ relaxation time percentage variation were calculated between pre- and post-contrast scans. Nanoparticle accumulation in tumors was assessed by comparing post-contrast T_2_ values and T_2_ variation between the FMX and FMX + FUS groups. Fold enhancement was calculated as the ratio of the median tumor transverse relaxation rate change (ΔR_2_) in the FMX + FUS group relative to the FMX group. The beam width of the intratumoral sonicated region was estimated from post-contrast T_2_-weighted images and compared with the corresponding beam widths measured in healthy mice subjected to the same FUS pressure (Fig. S15). Brain tissues were subsequently processed for histological evaluation.

### 4.14 Statistical analysis

All statistical analyses were performed using MATLAB (MathWorks). Data from simulations are presented as mean ± SD. Relaxivity values are reported as linear-fit slopes ± standard error of the fitted slope. For quantities displayed as boxplots and distributions, values are reported as median with interquartile range (IQR).

Longitudinal microbubble stability studies were evaluated using repeated-measures ANOVA. Comparisons of quantities (whole-brain fluorescence intensity ratio, T_2_ relaxation time, T_2_ relaxation time variation, fluorescence intensity ratios in histology slides, H&E damage score, beam width, threshold pressure) between independent groups of mice were performed using the two-tailed Wilcoxon rank-sum test. Comparisons of quantities (T_2_ relaxation time, normalized fluorescence intensity and cell density in histology slides) within the same mouse were performed using the Wilcoxon signed-rank test; P < 0.05 was considered statistically significant. For the clearance study *in vivo*, a one-way repeated-measures ANOVA was performed in MATLAB using *fitrm* and *ranova* to test for differences across four time points (1, 4, 8, and 24 h), where time was included as a within-subject factor.

The MATLAB *fitlm* function was used to fit linear regression models of FMX relaxation rate in phantoms as a function of iron concentration, simulated *in situ* peak negative pressure as a function of applied acoustic pressure, simulated beam width as a function of threshold pressure, experimental beam width as a function of the applied pressure, brain relaxation rate as a function of the administered FMX dose, and microbubble volume, concentration, and diameter as a function of time. P values for individual regressions were calculated from the null hypothesis that the slope was equal to zero. Differences between slopes of two regression lines (transverse relaxation rate of FMX vs. FMX-FITC as a function of iron concentration and brain transverse relaxation rate in FUS-treated vs. untreated regions as a function of administered dose) were evaluated by fitting a linear model to the difference between paired responses (ΔY) as a function of X and testing whether the slope of ΔY was significantly different from zero. Comparison of the regression line of the *in situ* peak negative pressure as a function of applied acoustic pressure to the identity line (y = x) was performed using a joint hypothesis test of slope = 1 and intercept = 0, with significance reported as the corresponding P value. Statistical significance in figures is indicated as ∗P < 0.05, ∗∗P < 0.01, and ∗∗∗P < 0.001.

## Funding

Eunice Kennedy Shriver National Institute of Child Health and Human Development Grant R01HD103638 (HED).

National Institute of Neurological Disorders and Stroke Grant UG3NS115637 (RDA).

Knut and Alice Wallenberg Foundation Postdoctoral Fellowship KAW 2023.0463 (GMS).

Foundation Blanceflor Scholarship 2024:1 (GMS).

MSCA Postdoctoral Fellowship 101,068,340 (GA).

## CRediT authorship contribution statement

**Giovanni M. Saladino:** Conceptualization, Data curation, Formal analysis, Funding acquisition, Investigation, Methodology, Project administration, Visualization, Writing – original draft, Writing – review & editing. **Payton J. Martinez:** Data curation, Formal analysis, Investigation, Methodology, Visualization, Writing – original draft, Writing – review & editing. **Jie Wang:** Investigation, Writing – review & editing. **Raheleh Roudi:** Investigation, Writing – review & editing. **Giacomo Annio:** Funding acquisition, Investigation, Writing – review & editing. **Raag D. Airan:** Funding acquisition, Supervision, Validation, Writing – review & editing. **Heike E. Daldrup-Link:** Conceptualization, Funding acquisition, Supervision, Validation, Writing – review & editing.

## Declaration of competing interest

The authors declare that they have no known competing financial interests or personal relationships that could have appeared to influence the work reported in this paper.

## Data Availability

Data will be made available on request.
