# Supplementary material for: Transient blood-brain barrier opening by focused ultrasound enhances ferumoxytol accumulation in glioblastoma
Source: Mater Today Bio. 2026 May 26;38:103280. doi: 10.1016/j.mtbio.2026.103280 (PMC13242036; doi:10.1016/j.mtbio.2026.103280)
Supplement: Multimedia component 1 [file mmc1.pdf]

Supplementary Material

Transient Blood-Brain Barrier Opening by Focused Ultrasound Enhances Ferumoxytol Accumulation in Glioblastoma

Giovanni M. Saladino,[1†*] Payton J. Martinez,[1†] Jie Wang,[1] Raheleh Roudi,[1] Giacomo Annio,[1] Raag D. Airan,[1‡] Heike E. Daldrup-Link[1‡]

*Corresponding author. Email: gmsaladino@stanford.edu
[†, ‡] These authors contributed equally to this work.

This PDF file includes:

Supplementary Text
Figs. S1 to S17
Tables S1

Supplementary Text

Fluorescein Labeling

UV-vis absorption spectra of FMX and FMX-FITC are shown in **Fig. S2A**. Subtraction of the normalized FMX spectrum from that of FMX-FITC revealed an absorption band centered at 495 nm (**Fig. S2B**). One-dimensional fluorescence measurements confirmed an emission peak at 520 nm upon excitation at 495 nm (**Fig. S3A**) and an excitation peak at 495 nm when monitoring emission at 520 nm (**Fig. S3B**).

FITC conjugation did not alter the dry core size or morphology of FMX, as shown by TEM and size-distribution analysis. The slight increase in hydrodynamic diameter observed by DLS is consistent with the presence of an added organic layer and associated changes in surface hydration. The optical measurements confirmed the presence of fluorescein on FMX, with absorption and emission maxima matching those of free FITC. The preservation of these spectral features indicates that the fluorophore remains optically active after conjugation, enabling fluorescent detection of FMX-FITC.

The similar relaxivities of FMX and FMX-FITC in tissue-mimicking phantoms indicated that fluorescent labeling does not substantially affect the magnetic properties responsible for T2 contrast. Together, these results support the use of FMX-FITC as a dual-mode nanoparticle for MRI and optical fluorescence studies.

Focused Ultrasound Simulations

The k-Wave simulation framework was first validated against experimental hydrophone calibrations of the 650 kHz single-element FUS transducer in water (**Fig. S4A**, left). Simulated pressure fields closely matched experimentally measured beam profiles across imaging planes (**Fig. S4A**, right). Quantitative comparison between simulated and measured pressure fields in the XZ plane yielded a root mean square error (RMSE) of 0.0711, a mean absolute error (MAE) of 0.0543, and a coefficient of determination (R^2) of 0.8899 (**Fig. S4B**). Similarly, comparison in the XY plane resulted in an RMSE of 0.0849, an MAE of 0.0780, and an R^2 of 0.9292 (**Fig. S5**), confirming accurate reproduction of the focal geometry and spatial pressure distribution.

To interpret the *in vivo* delivery results and guide parameter selection, we developed and validated a simulation framework describing FUS propagation and beam geometry under the experimental conditions used in our workflow. Quantitative validation against hydrophone measurements in water demonstrated strong agreement between simulated and experimental pressure fields, supporting the accuracy of the k-Wave model for predicting focal geometry and spatial pressure distribution. Minor oscillatory features were observed along the axial (z) direction in the experimental measurements that were not fully captured in the simulations. These features are consistent with standing-wave artifacts introduced by hydrophone reflections during water-tank measurements rather than intrinsic characteristics of the simulated acoustic field [27].

Microbubble Preparation and Stability

Definity microbubbles were prepared immediately prior to characterization and administration. Specifically, microbubbles were generated by mechanical agitation for 45 s using a dental amalgamator (Lantheus Medical Imaging), producing perflutren lipid microspheres. The activated MBs were stored immediately after formation and injected within 6 h of activation.

Microbubble (MB) concentration and number- and volume-weighted size distributions were measured using a Multisizer 3 (Beckman Coulter). MB concentration (MBs/mL) was plotted as a function of MB volume (µL), and the MB gas volume fraction (Φ) was calculated as follows:

$$\phi MB = \sum_{i=1}^{n} v_i \times c_i$$

where i denotes the sizing-bin index across 300 bins spanning diameters of 0.7-30 µm. MB preparations were characterized 6 h prior to focused ultrasound (FUS) treatment to verify size distribution and concentration. MBs were stored at 4 °C until use and diluted to the injection concentration within 5 min before administration. Microbubble size distributions measured 1 h after preparation, after 4 h of storage at 4 °C, and 1 week after reconstitution are shown as number-weighted and volume-weighted distributions in **Fig. S8A-C**. Across all time points, microbubbles exhibited similar size profiles, with diameters of 1.52 µm (IQR 0.03 µm) at 0 h, 1.62 µm (IQR 0.16 µm) at 4 h, and 1.68 µm (IQR 0.01 µm) at 1 week. Repeated-measures ANOVA showed no significant difference in microbubble size across the used conditions for animal experiments ($P = 0.1357$; **Fig. S8D**).

The relationship between microbubble concentration and bubble volume for the stock formulation is shown in **Fig. S9A**, with the shaded area representing the total administered microbubble volume. The injected microbubble volume dose was consistent across animals, using 0.5 µL/g per mouse, 9.5 (IQR 3.93) uL/kg microbubble volume dose, **Fig. S9B**). Representative bright-field microscopy visually confirmed the spherical morphology and polydisperse size range of the microbubbles (**Fig. S9C**).

Microbubble stability over time was further evaluated by monitoring total microbubble volume, concentration, and mean diameter from the same stock (**Fig. S10A-C**). Linear regression analysis revealed no significant time-dependent changes in microbubble volume ($R^2 = 0.09$, $P = 0.39$), concentration ($R^2 = 0.20$, $P = 0.19$), or mean diameter ($R^2 = 0.15$, $P = 0.27$), indicating stable microbubble properties over time.

Microbubble characterization provides context for interpreting the FUS experiments. The observed stability of microbubble size, concentration, and total gas volume across storage conditions and over the experimental time window indicates that the cavitation environment was similar between animals and imaging sessions. Because microbubble dynamics influence the extent of BBB opening [29], this stability supports the attribution of differences in FMX delivery and MRI contrast primarily to ultrasound exposure and tissue properties rather than to variability in the microbubble formulation. The reproducibility of the administered microbubble volume dose further supports quantitative comparisons across experimental groups.

Validation Study with Gadobutrol

To validate FUS-mediated BBB opening at 650 kHz using a clinically employed molecular MRI contrast agent, healthy CD-1 mice were treated with Definity microbubbles followed by FUS treatment and intravenous administration of gadobutrol (GDB; Gadavist®; 0.3 mmol/kg). T1-weighted MRI showed localized GDB enhancement within the sonicated brain region, confirming focal BBB opening and delivery of a small molecular probe (**Fig. S13A**). Quantification of the contrast-enhanced region showed a measurable beam width in healthy brain tissue (**Fig. S13B**). Compared with FMX delivery in healthy mice (**Fig. 6**), GDB produced a significantly larger beam width, consistent with broader distribution of the smaller molecular probe compared with nanoparticle formulations. The median beam width differed significantly between GDB and FMX, with GDB producing a larger beam width than FMX (3.08 mm, IQR = 0.44, N = 4 *vs.* 2.32 mm, IQR = 0.19, N = 5; P = 0.016). The estimated opening threshold pressure was significantly lower for GDB than for FMX, with median values of 0.144 MPa for GDB (IQR = 0.028, N = 4) and 0.265 MPa for FMX (IQR = 0.016, N = 5; P = 0.016; **Fig. S13C**). These results provided an independent validation of FUS-mediated BBB opening using a molecular probe under the same 650 kHz ultrasound conditions.

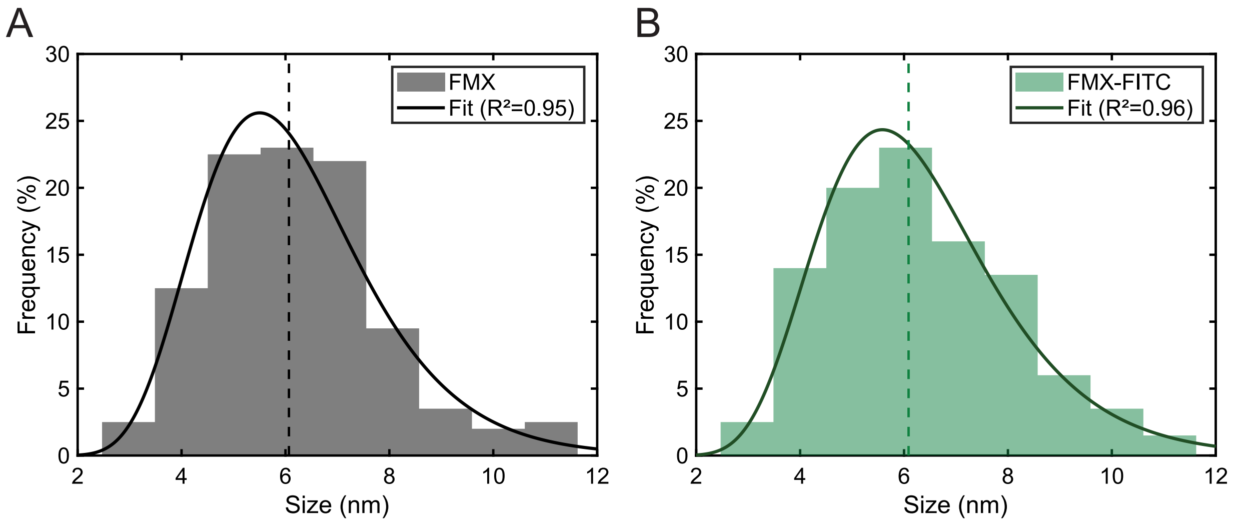

Fig. S1.

Nanoparticle size distributions. (**A**) Core diameter histogram of FMX (N = 200, Bin Width = 1 nm) measured by transmission electron microscopy (TEM) and fitted with a lognormal distribution (black line, R^2 = 0.95). The median value (6.07 nm) is indicated with a dashed black line. (**B**) Core diameter histogram of FMX-FITC (N = 200, Bin width = 1 nm) measured by TEM and fitted with a lognormal distribution (green line, R^2 = 0.96). The median value (6.09 nm) is indicated with a dashed green line.

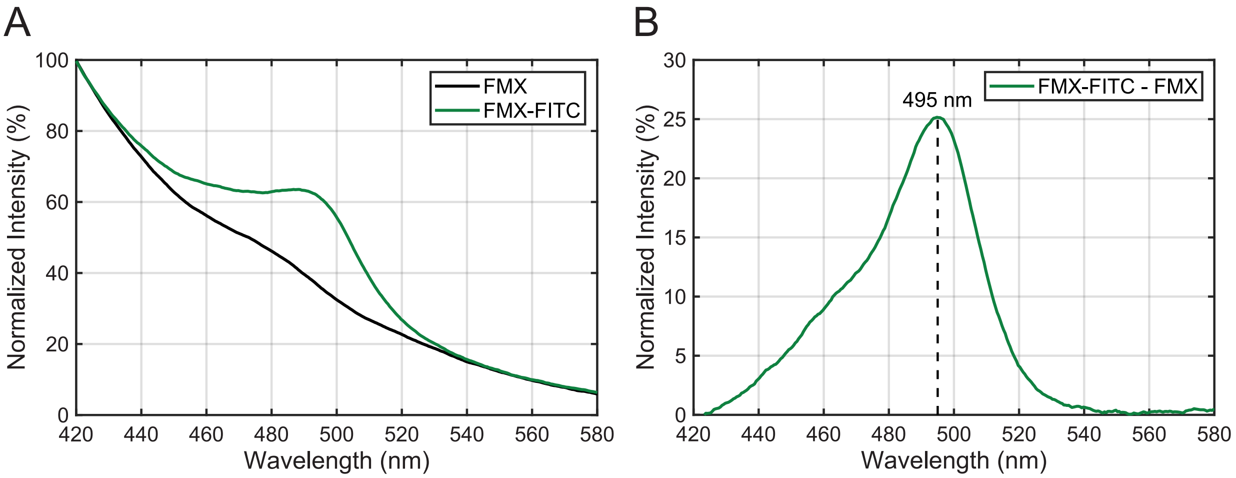

Fig. S2.

Absorption Spectroscopy Analysis. (**A**) UV-vis absorption spectra of FMX (black) and FMX-FITC (green). (**B**) Difference between the normalized UV-vis spectra of FMX-FITC and FMX, highlighting the FITC-specific absorbance contribution, with the characteristic fluorescein absorption band after conjugation peaked at 495 nm.

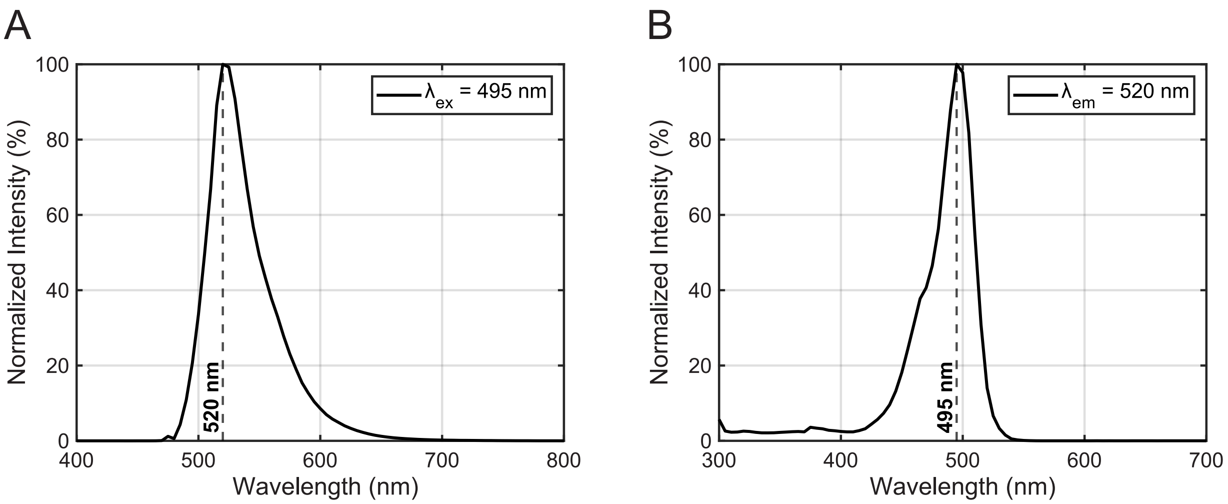

Fig. S3.

Fluorescence Spectroscopy Analysis. (**A**) Fluorescence emission spectrum of FMX-FITC measured using an excitation wavelength of 495 nm, showing a maximum emission peak at 520 nm (grey dashed line). (**B**) Fluorescence excitation spectrum of FMX-FITC recorded by monitoring emission at 520 nm, showing a maximum excitation peak at 495 nm (grey dashed line).

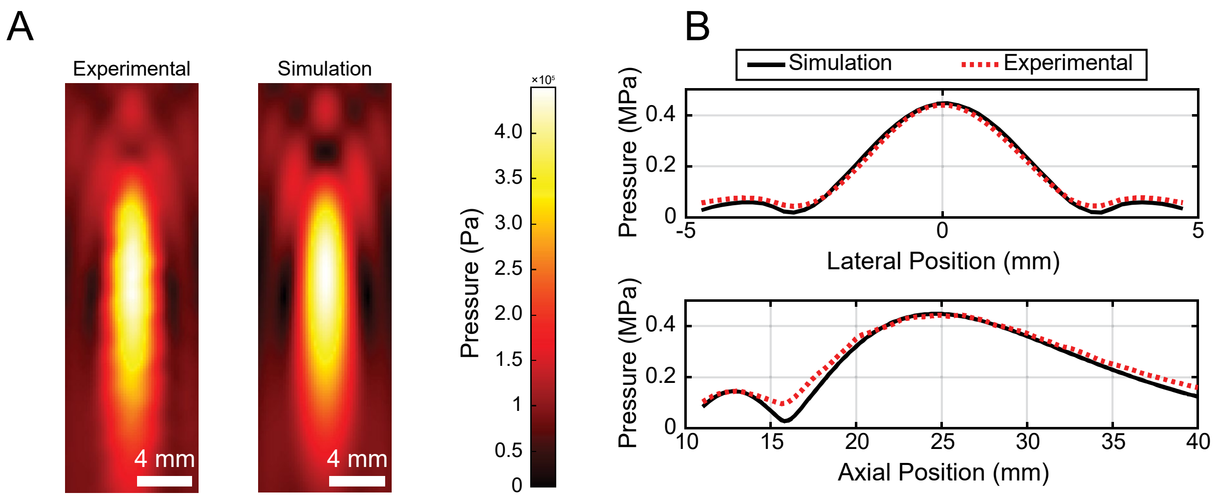

Fig. S4.

Validation of k-Wave simulations in the Sagittal (XZ) Plane. (**A**) Experimentally measured (left) and simulated (right) peak negative pressure fields of the 650 kHz single-element focused ultrasound (FUS) transducer in water. The color bar denotes peak negative pressure values, and the scale bar represents 4 mm. (**B**) Quantitative comparison between simulated (black) and experimental (red) pressure fields in water in the XZ plane, showing strong agreement in focal geometry and spatial pressure distribution (RMSE = 0.0711, MAE = 0.0543, R^2 = 0.8899).

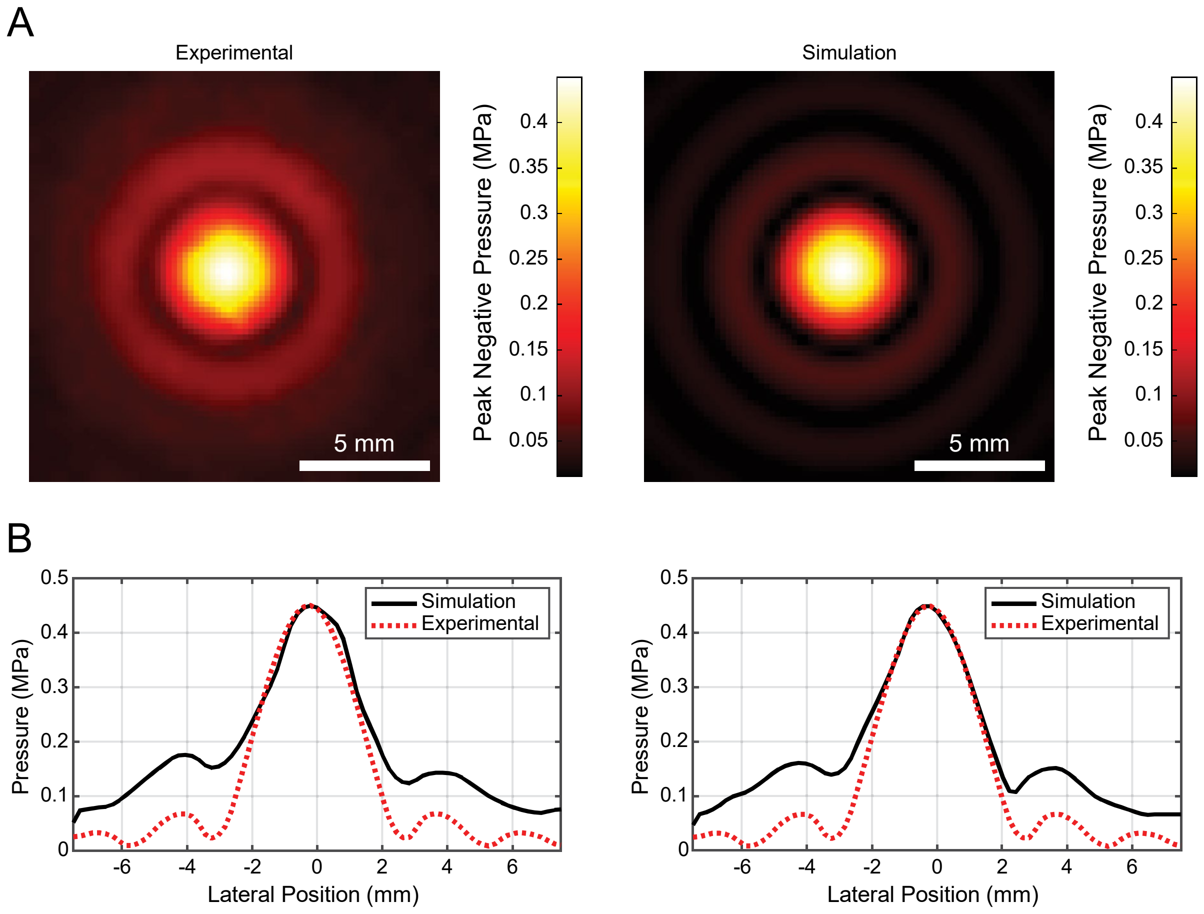

Fig. S5.

Validation of k-Wave simulations in the transverse (XY) plane. (**A**) Experimentally measured (left) and simulated (right) peak negative pressure fields of the 650 kHz single-element focused ultrasound (FUS) transducer in water. The color bar denotes peak negative pressure values, and the scale bar represents 2 mm. (**B**) Quantitative comparison between simulated (black) and experimental (red) pressure profiles along the two orthogonal centerlines in the XY plane (left–right and anterior–posterior), showing strong agreement in focal geometry and spatial pressure distribution (RMSE = 0.0849, MAE = 0.0780, R^2 = 0.9292).

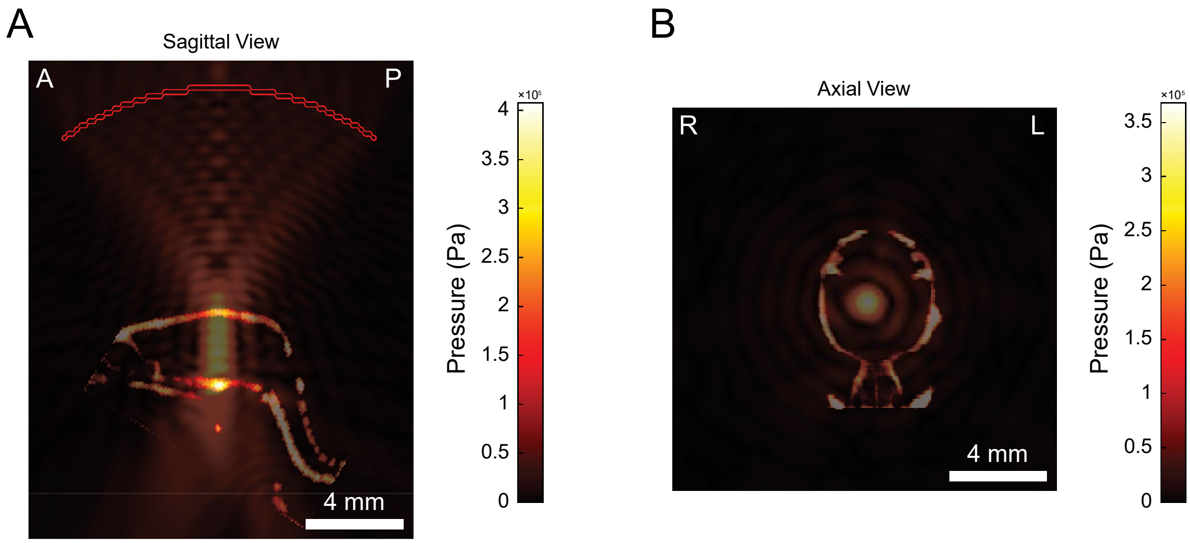

Fig. S6.

Focused Ultrasound Simulations. (**A**) Sagittal slice through the acoustic axis showing simulated peak negative pressure overlaid on the mouse brain CT used to define geometry and acoustic properties. (**B**) Axial slice at the focal plane showing the corresponding pressure field. Color bar indicates pressure, and the scale bar is 4 mm.

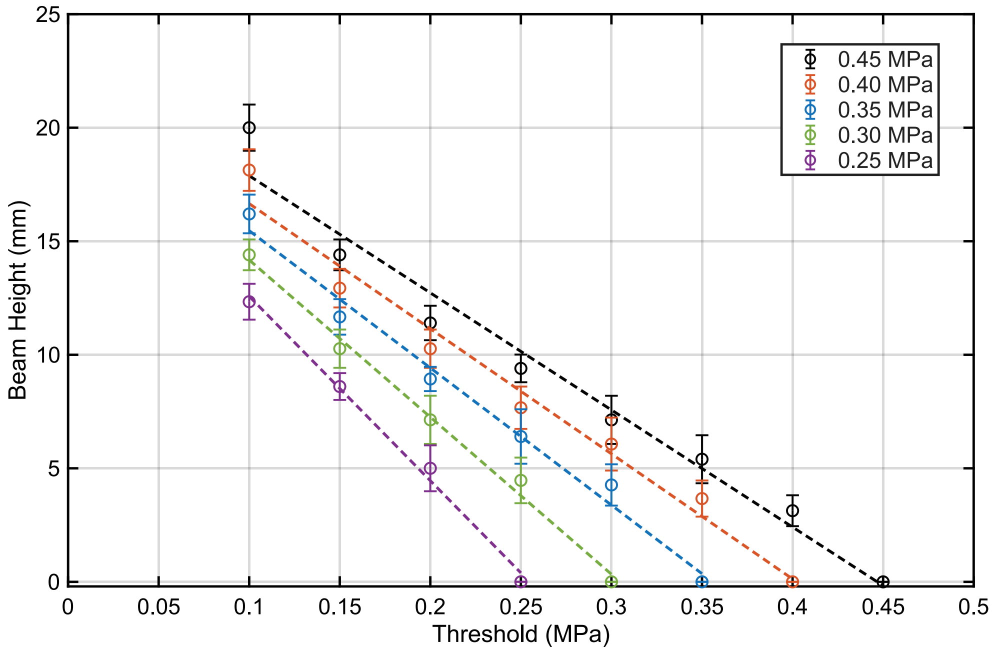

Fig. S7.

Simulated Beam Height. Estimated beam height as a function of threshold pressure for five simulated acoustic pressure levels. Dashed lines of the corresponding colors indicate linear regression fits for each simulated pressure condition, with $R^2 > 0.97$ and $P < 0.005$ for all fits.

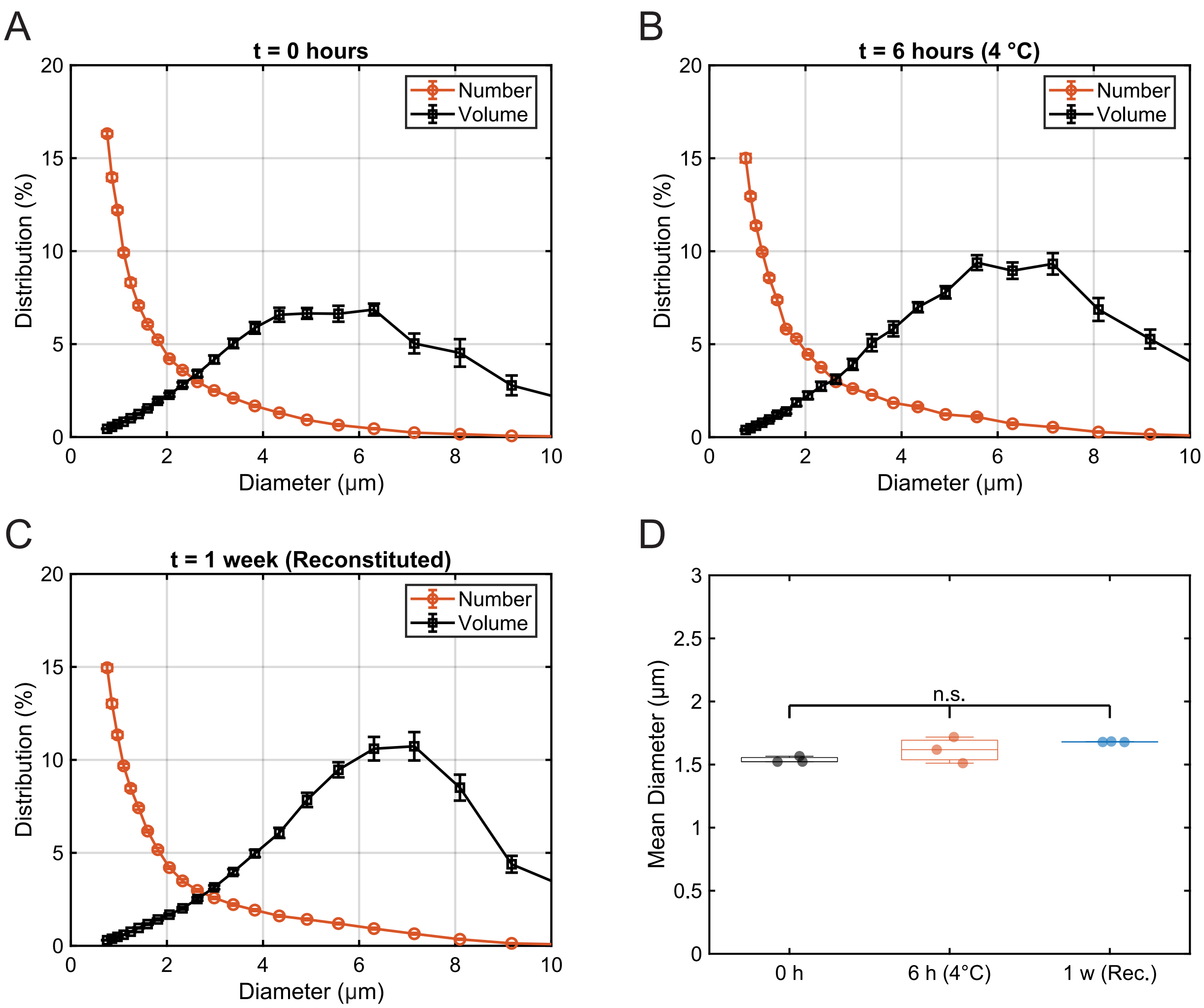

Fig. S8.

Microbubble Characterization. Distributions of microbubble diameter measured at (**A**) 1 h after preparation, (**B**) after 4 h storage at 4 °C, and (**C**) 1 week (sample reconstituted 10 minutes before measurement). Number-weighted distributions are shown in orange and volume-weighted distributions in black. (**D**) Scatter box plot of mean microbubble diameter for each condition (N = 3 independent preparations), showing no significant difference over time (repeated measures ANOVA, $P > 0.05$).

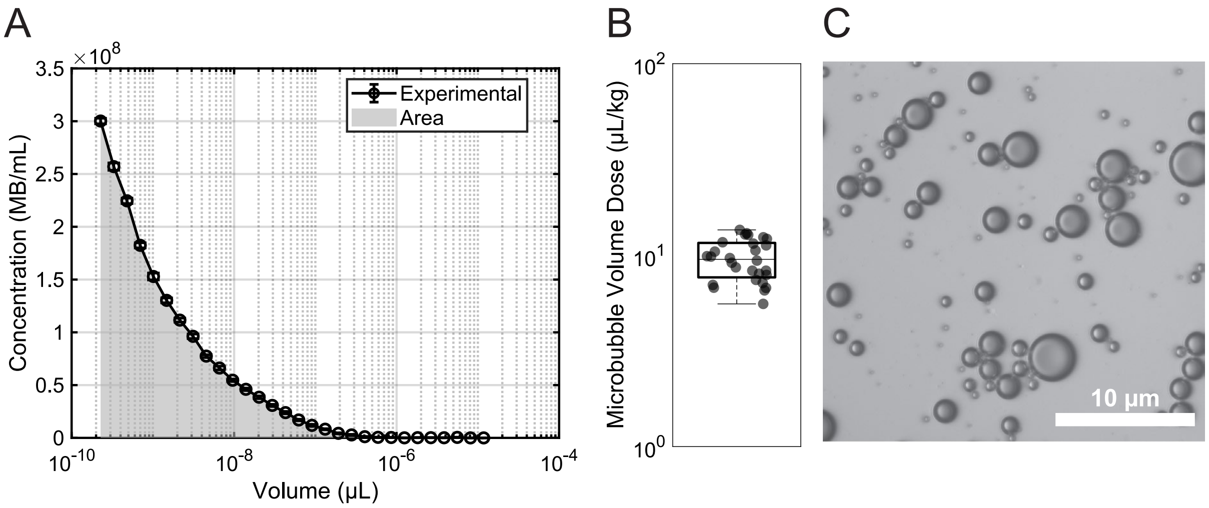

Fig. S9.

Microbubble Dose Evaluation. (**A**) Microbubble concentration versus bubble volume for the stock formulation of Definity microbubbles, with the area under the curve (shaded in grey) representing the gas volume fraction total microbubble volume (μL/mL), measured via Coulter Counter. (**B**) Scatter-box plot of microbubble volume dose administered to the injected mice. Total microbubble gas volume administered per mouse (μL/kg), shown as a scatter box plot with each point representing one mouse (N = 28). (**C**) Representative bright-field micrograph of the microbubbles in suspension (stock concentration, 2×10^9 MB/mL), with a 10 μm scale bar.

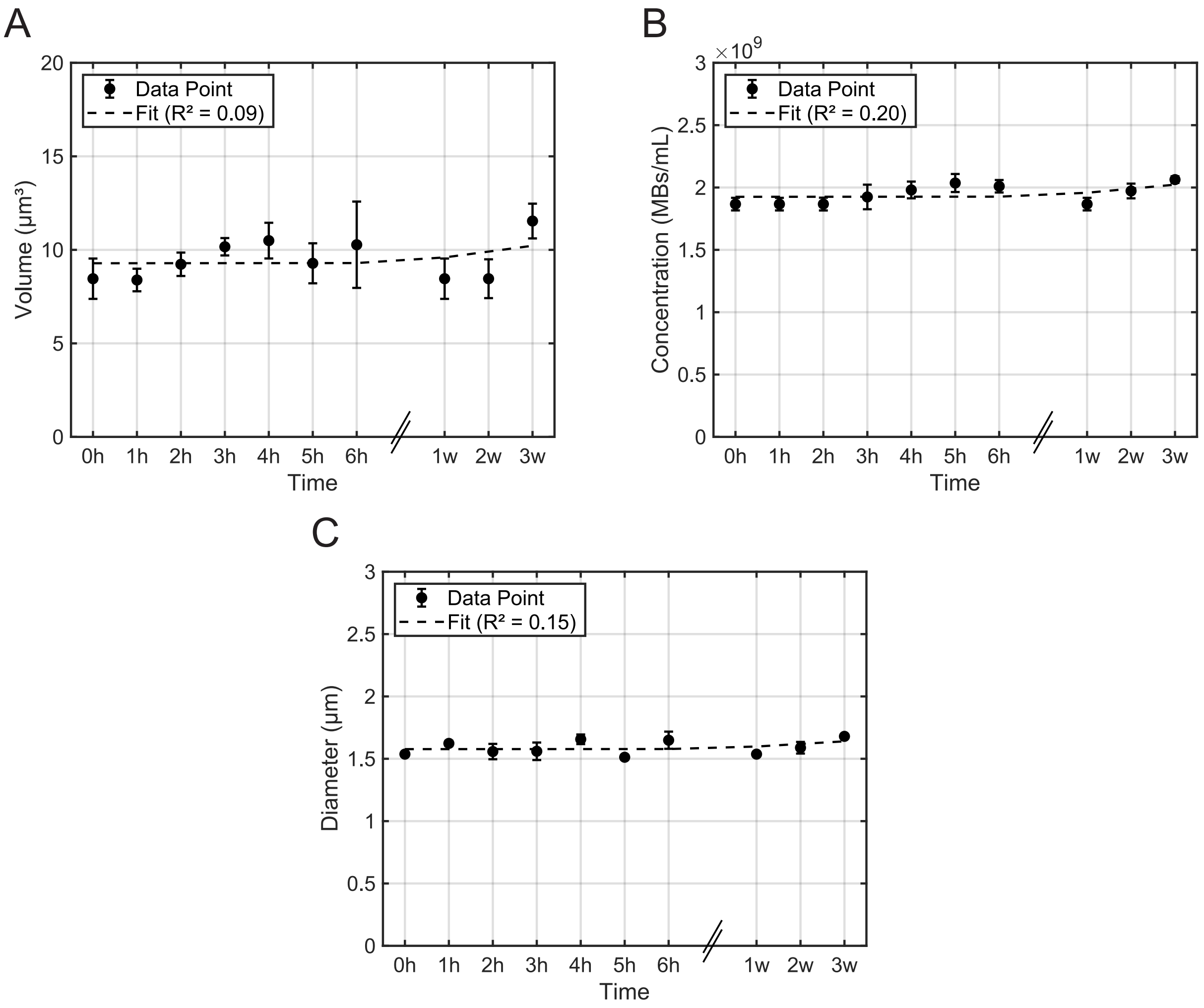

Fig. S10.

Microbubble Stability Study. (**A**) Total microbubble gas volume from a single stock preparation measured at indicated time points under storage conditions, with a linear fit (1.86×10^{-3} μm^3 h^{-1}, $R^2 = 0.09$, P = 0.39). (**B**) Microbubble concentration (MBs per mL) over time with linear fit (2×10^5 MBs mL^{-1} h^{-1}, $R^2 = 0.20$, P = 0.19). (**C**) Mean microbubble diameter (micrometers) over time with linear fit (1.24×10^{-4} μm h^{-1}, $R^2 = 0.15$, P = 0.27). Points represent repeated measurements from the same stock (N = 10 time points, 3 technical replicates). Slopes were not significantly different from zero for any parameter (P > 0.05).

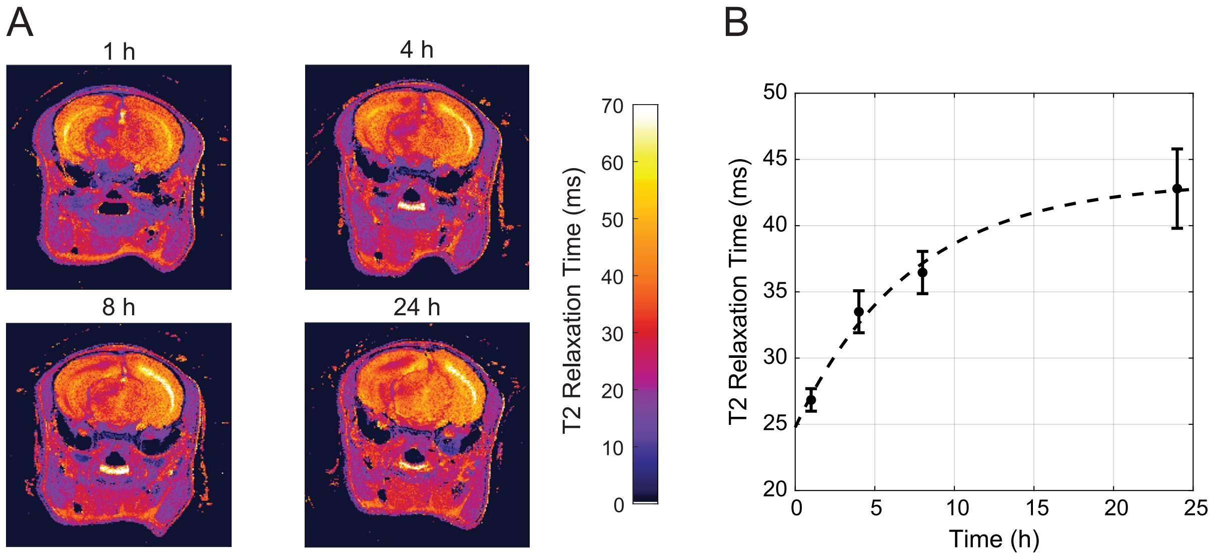

Fig. S11.

Clearance Study. (**A**) Representative T2 maps of the brain acquired at 1, 4, 8, and 24 h after focused ultrasound treatment and administration of ferumoxytol (FMX, 30 mgFe/kg), showing progressive recovery of relaxation time over time. (**B**) Plot of T2 relaxation time as a function of time in the sonicated region, showing exponential recovery within the first 24 h (dashed line, R^2 = 0.990, P < 0.001, N = 5).

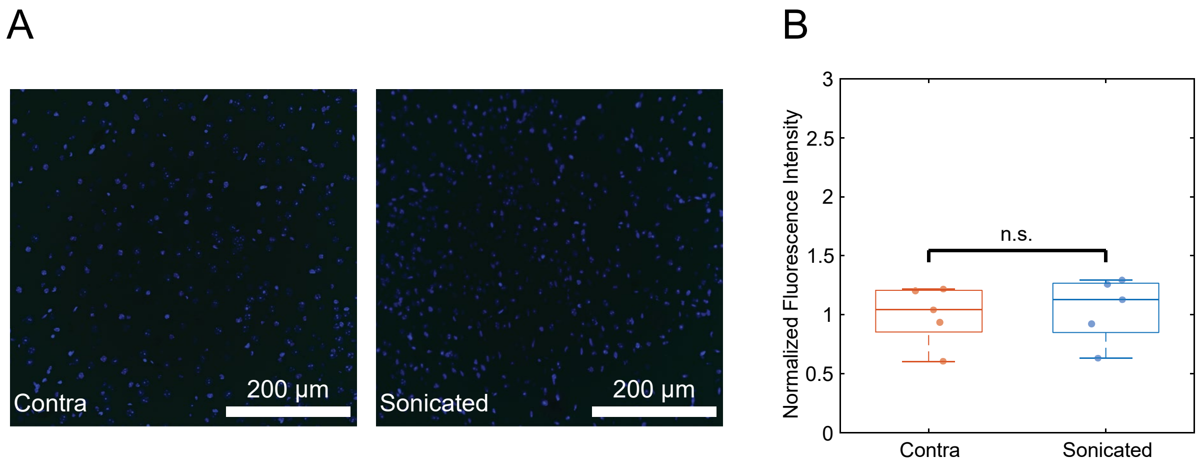

Fig. S12.

Assessment of Acute Neurodegeneration. (**A**) Representative Fluoro-Jade C-stained brain tissue slides acquired 24 h after FMX administration and microbubble-mediated FUS treatment, showing no detectable Fluoro-Jade C-positive neurons in the sonicated brain region. (**B**) Scatter-box plot of normalized green fluorescence intensity in the sonicated and contralateral hemispheres, showing no significant difference between regions (N = 5).

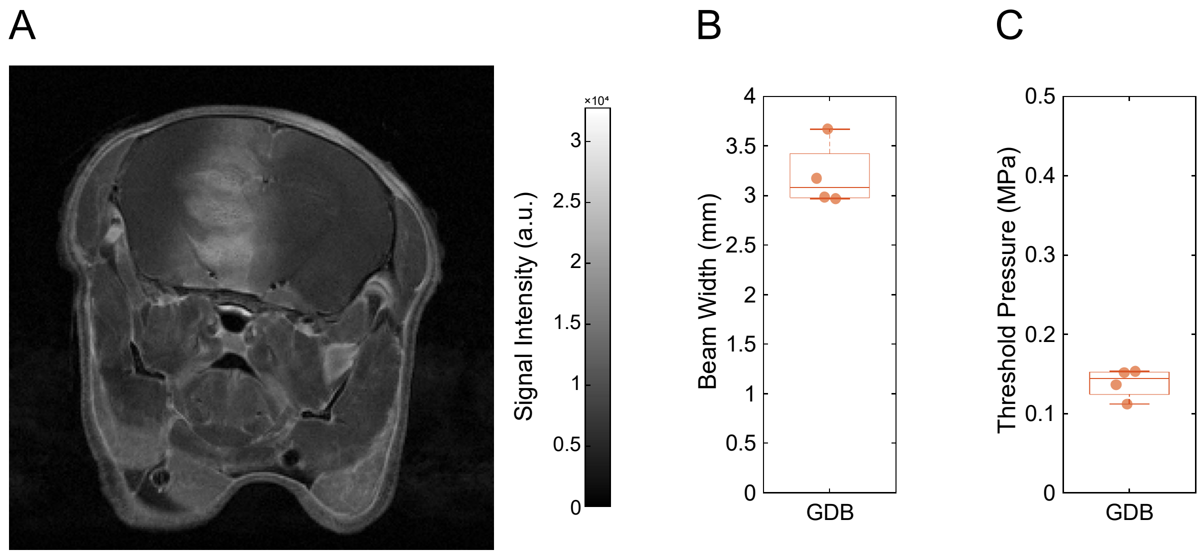

Fig. S13.

FUS-mediated Delivery of Gadobutrol. (**A**) T1-weighted brain MR image of a healthy mouse after focused ultrasound treatment and GDB administration (0.3 mmol/kg). (**B**) Scatter-box plot of the quantified contrast-enhanced beam width in healthy tissue (N = 4). (**C**) Scatter-box plot of the estimated BBB opening threshold pressure in healthy tissue (N = 4).

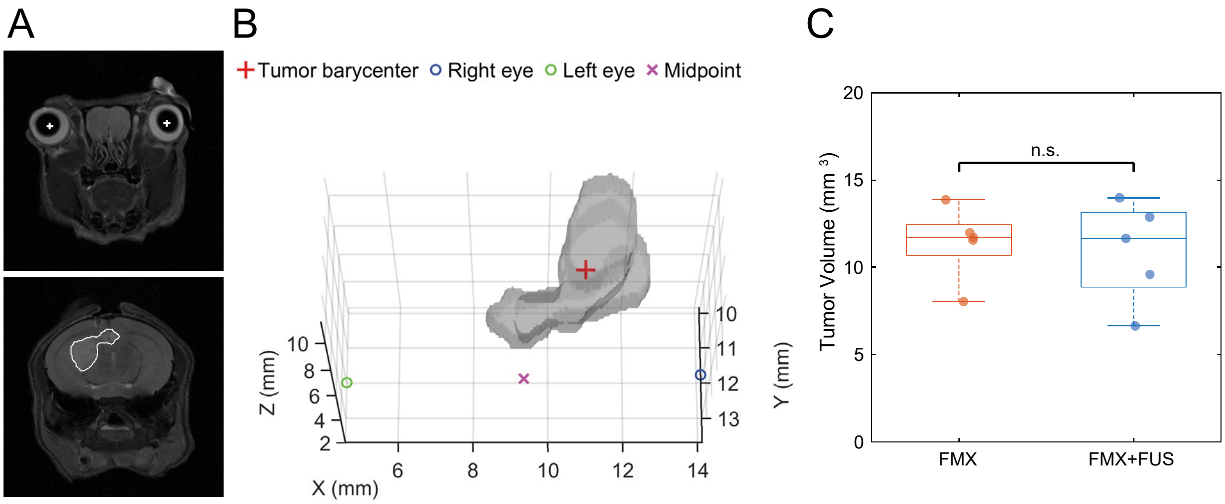

Fig. S14.

MRI-based Tumor Localization and Volume Estimation. (**A**) Coronal T2-weighted MRI slices (2D TurboRARE sequence, voxel size = 70 × 70 × 700 μm^3) at the level of the eyes, with eye centers indicated by white dots as reference points, and at the tumor level, showing manual polygon segmentation, reconstructed into 3D volumes by spline interpolation along Z. (**B**) Three-dimensional rendering of the tumor mask with left and right eye centers, their midpoint, and the tumor barycenter, used to estimate tumor position relative to the eye midpoint. (**C**) Scatter-box plot of estimated tumor volumes for the FMX and FMX+FUS groups, showing no significant difference between groups (P = 1, N = 5 per group).

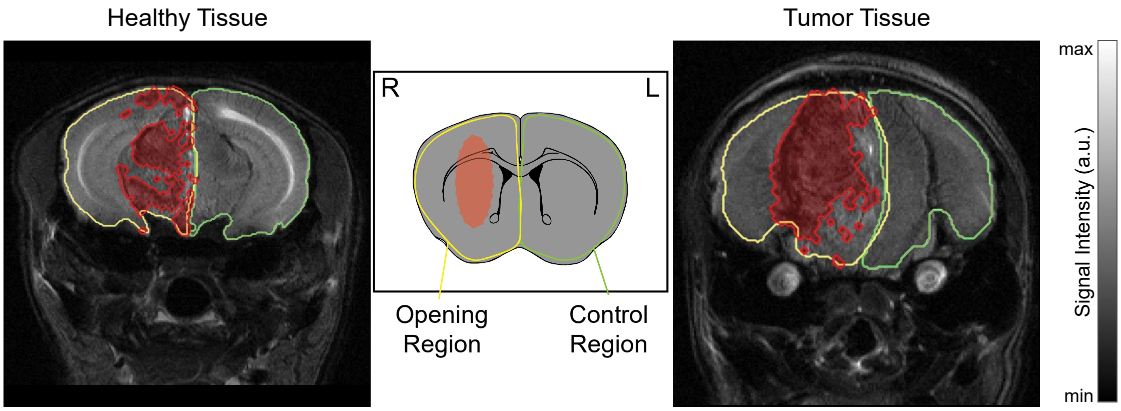

Fig. S15.

Significant BBB Opening Region Determination. ROIs were selected in the control region (green), the mean and standard deviation was determined, and a threshold was defined by mean - 2 standard deviations. The BBB opening region was defined (yellow) and all pixels below this threshold were found to be significant BBB opening (red). Representative images are shown for healthy (left) and tumor bearing (right) mice.

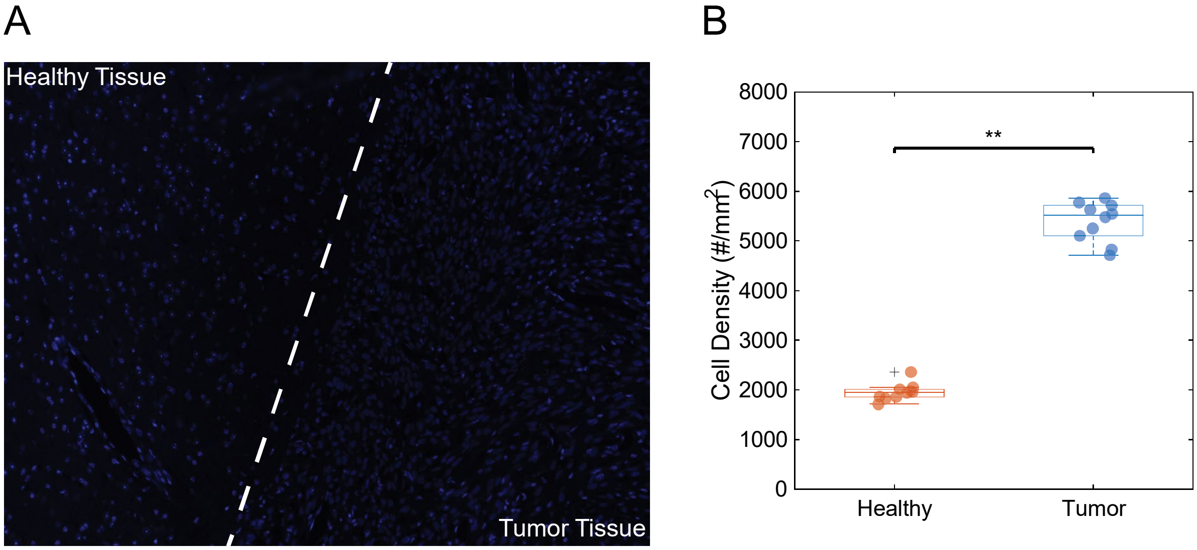

Fig. S16.

Cell Density Analysis. (**A**) Representative fluorescence image of a DAPI-stained brain histology section showing the interface between healthy tissue and tumor tissue. (**B**) Scatter-box plot comparing cell density in healthy and tumor tissue, highlighting a significant statistical difference between the two regions ($P = 0.002$, $N = 10$ per group).

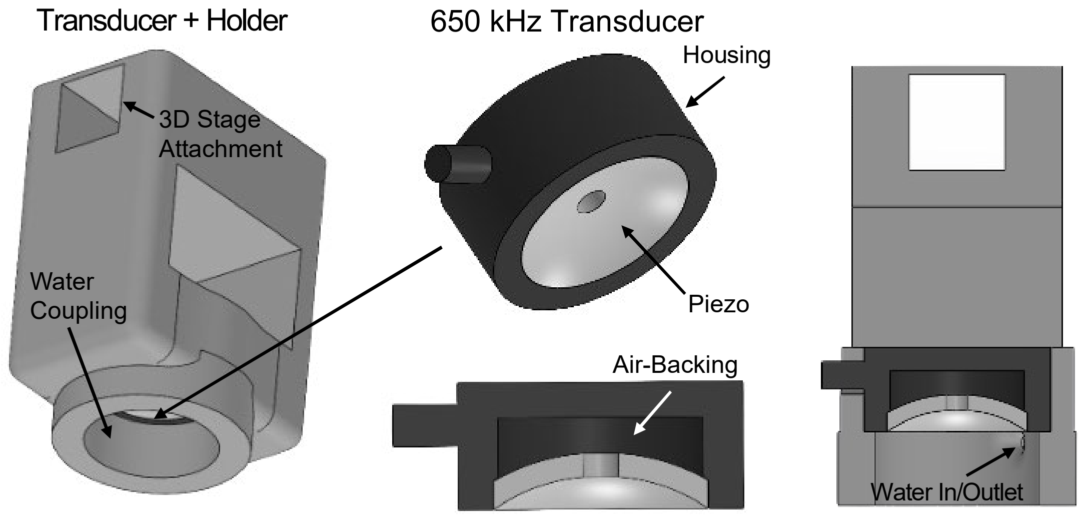

Fig S17.

Custom FUS Transducer Holder and Coupling Assembly. Three-dimensional CAD rendering of the assembled 650 kHz single element transducer mounted in a custom water filled holder for transcranial sonication. The holder provides an acoustic coupling path to the mouse scalp via an acoustic window and mounts to a 3D positioning stage through the stage interface. Labeled features include the water coupling chamber, acoustic window, fill port or seal, and stage mounting interface. Stage attachment was made using PLA, transducer housing was made from ABS-like (Watershed) material, and the piezo element is PZT-4.

Table S1.
H&E Histopathology Grading Rubric. Rubric used for H&E analysis following focused ultrasound–mediated BBB opening. The standardized scoring criteria assess red blood cell (RBC) extravasation and microhemorrhage [51], vascular and endothelial alterations [51], parenchymal vacuolation/edema [52], and neuronal injury [51]. This rubric has been previously applied for evaluation of BBB opening in both tumor and healthy brain tissue.

Category	Severity Definition	Representative Criteria
RBC extravasation / Micro-hemorrhage	0 = none; 1 = rare perivascular RBCs; 2 = multiple petechial foci; 3 = confluent/multi-vessel; 4 = large/widespread hemorrhage	Extravascular erythrocytes, perivascular bleeding, parenchymal pooling
Vascular / Endothelial Changes	0 = normal; 1 = mild dilation or perivascular space; 2 = clear dilation, mild wall change; 3 = many vessels, enlarged perivascular space; 4 = widespread distortion	Enlarged capillaries, perivascular cuffs, structural vessel disruption
Parenchymal Vacuolation / Edema	0 = none; 1 = tiny focal vacuoles; 2 = vacuolation in ≤25% of ROI; 3 = confluent vacuolation in 25–50%; 4 = extensive spongiosis/edema affecting >50%	Micro-vacuoles in neuropil, spongy/parenchymal clearing
Neuronal Injury (Eosinophilic / Pyknotic Cells)	0 = normal neurons; 1 = rare degenerating neurons; 2 = increased focal degeneration; 3 = multifocal clusters; 4 = widespread necrosis	Shrunken, eosinophilic neurons, pyknotic nuclei
Composite BBB Damage Score	Sum of above (0–16)	Summed pathology across categories
